# Digital gene expression analysis of the response to *Ralstonia solanacearum* between resistant and susceptible tobacco varieties

**DOI:** 10.1038/s41598-021-82576-8

**Published:** 2021-02-16

**Authors:** YanYan Li, Lin Wang, GuangWei Sun, XiHong Li, ZhenGuo Chen, Ji Feng, Yong Yang

**Affiliations:** 1 Tobacco Research Institute of Hubei Province, Wuhan, 430030 China; 2 China Tobacco Hubei Industrial Co., Ltd., Wuhan, 430040 China; 3 School of Life Sciences, Hubei University, Wuhan, 430062 China

**Keywords:** Plant sciences, Plant breeding, Plant stress responses

## Abstract

Tobacco bacterial wilt (TBW) caused by *Ralstonia solanacearum* is the most serious soil-borne disease of tobacco. However, molecular mechanism information of *R. solanacearum* resistance is limited to tobacco, hindering better breeding of resistant tobacco. In this study, the expression profiles of the rootstalks of Yunyan87 (susceptible cultivar) and Fandi3 (resistant cultivar) at different stages after *R. solanacearum* infection were compared to explore molecular mechanisms of tobacco resistance against the bacterium. Findings from gene-expression profiling indicated that the number of upregulated differentially expressed genes (DEGs) at 3 and 7 days post-inoculation (dpi) increased significantly in the resistant cultivar. WRKY6 and WRKY11 family genes in WRKY transcription factors, ERF5 and ERF15 family genes in ERFs transcription factors, and genes encoding PR5 were significantly upregulated in the resistant cultivar response to the infection. For the first time, *WRKY11* and *ERF15* were found to be possibly involved in disease-resistance. The Kyoto Encyclopedia of Genes and Genomes analysis demonstrated glutathione metabolism and phenylpropane pathways as primary resistance pathways to *R. solanacearum* infection. In the resistant cultivar, DEGs encoding CYP450, TCM, CCoAOMT, 4CL, PAL, CCR, CSE, and CADH, involved in the synthesis of plant antitoxins such as flavonoids, stilbenoids, and lignins, enriched in the phenylpropane pathway were upregulated at 3 and 7 dpi. Furthermore, a pot experiment was performed to verify the role of flavonoids in controlling TBW. This study will strongly contribute to a better understanding of molecular interactions between tobacco plants and *R. solanacearum*.

## Introduction

*Ralstonia solanacearum* is an important phytopathogen that attacks *Solanaceae* crops such as tomato, pepper, eggplant, tobacco, etc., worldwide. The disease caused by the pathogen is notoriously known as bacterial wilt with the symptom of wilting leaves or stems, vascular browning, or even death^1^. Tobacco bacterial wilt (TBW), caused by *R. solanacearum*, is the most serious soil-borne disease in tobacco plants. Most tobacco-growing countries with moist tropical or warm-temperate climates have a greater burden of this disease^2^. In China, the disease incidence has been gradually increasing, reaching up to 15–35%^3^. The increasing incidence is seriously threatening tobacco production in the 4 major tobacco-growing areas, including 14 provinces^4^. Thus, breeding resistant variety is considered an economical and effective measure to prevent TBW.

It is necessary to understand plant disease-resistance’s molecular mechanisms for breeding resistant variety, especially molecular breeding. Transcriptome analysis is useful to explore the molecular basis and reveal genes related to bacterial wilt resistance. For exploring the molecular mechanisms of potato-*R. solanacearum* interactions, 302 differentially expressed genes were identified by combining suppression subtractive hybridization and macroarray hybridization. Among them, 81 differentially expressed genes were considered *R. solanacearum* resistance-related genes, and these genes played putative roles in pathogen recognition, signal transduction, transcription factor functioning, hypersensitive response, systemic acquired resistance, cell rescue, and protection^5^. Through the transcriptome analysis of resistant and susceptible tomato cultivar after inoculation with *R. solanacearum*, more than 140 genes related to pathogenesis, hormone signaling, and lignin biosynthesis were increased in resistant cultivar, but no change found in susceptible cultivar^6^. Chen et al.^7^ performed global transcriptome profiling on resistant and susceptible peanut roots under *R. solanacearum* infection. They found that many unique genes were mainly involved in phytoalexins’ biosynthesis, particularly in the biosynthetic pathways of terpenoids and flavonoids. However, few studies have focused on transcriptomics of *R. solanacearum* resistance in tobacco, limiting its molecular information.

In order to resist the invasion of pathogens, plants have developed a sophisticated two-branch innate immune system. For the first branch of plant immunity, pattern-recognition receptors (PRRs) localized on the host cell surface recognize pathogen-associated molecular patterns (PAMPs) to activate PAMP-triggered immunity (PTI). However, pathogens release effectors inside the plant cell to suppress PTI. Thus the second branch of plant immunity, effector-triggered immunity (ETI) as the final defense strategy against pathogens, has been evolved in plants to recognize these effectors by nucleotide binding leucine-rich repeat (NB-LRR) resistance proteins^8–10^. In PTI, WRKY transcription factors (WRKY TFs) and ethylene-responsive TFs (ERF TFs) play important roles as regulators of plant immunity against phytopathogen, which are regulated by signaling synergistically mediated by ethylene (ET), jasmonic acid (JA), and salicylic acid (SA) signaling pathways^7,11–23^.

In this study, we used digital gene expression (DGE) profile analysis to identify and analyze gene-expression profiles of resistant and susceptible tobacco varieties infected with *R. solanacearum* for days 1, 3, and 7. We also screened resistance-related genes and pathway responses to *R. solanacearum*. This study will provide important insights into tobacco-*R. solanacearum* interactions, which is conducive to understand the molecular mechanisms of resistance responses to *R. solanacearum* in more *Solanaceae* crops.

## Materials and methods

### Tobacco materials and *R. solanacearum* inoculation

Tobacco cultivars Fandi3 (resistant to TBW, R) and Yunyan87 (susceptible to TBW, S) were provided by the Tobacco Research Institute of Hubei, Wuhan, China. The *R. solanacearum* strain HBLC5, which is highly pathogenic, was isolated from tobacco plants collected from tobacco plantations of Lichuan, Hubei, China^4^.

The seeds of resistant and susceptible cultivars were cultured in a greenhouse. When seedlings grew to the 5–6 leaf stage, the wounded roots of resistant and susceptible cultivars were inoculated with HBLC5 suspension (1 × 10^8^ CFU/mL) (OD600 = 0.1), according to the method suggested by Cellier and Prior^24^. The inoculated tobacco seedlings were cultured in a greenhouse at 28 ± 2 °C and a humidity of 95%. Symptoms were monitored at 1, 3, 7, and 25 days post-inoculation (dpi) using the scale described by Li et al.^4^. The incidence (*I*) and disease index (*DI*) of TBW were calculated using the following formula: *I* (%) = *n*/*N* × 100%, *DI* = 100 × (n1 × 1 + n2 × 3 + n3 × 5 + n4 × 7 + n5 × 9)/(*N* × 9), where *n* is the number of plants with symptoms of bacterial wilt; n1, n2, n3, n4, n5 are the number of plants with symptoms 1, 3, 5, 7, and 9, respectively; and *N* is the total number of observations.

### Sample collection

The stem base (0–1 cm above the root) of tobacco seedlings were sampled at 1, 3, and 7 dpi. The mock-inoculated tobacco seedlings of resistant and susceptible cultivars were used as controls and also sampled at the same time points. The samples were categorized into 4 groups: mock-inoculated samples of S (M-S), denoted as M-S1, M-S3, and M-S7; pathogen-inoculated samples of S (In-S), denoted as In-S1, In-S3, and In-S7; mock-inoculated samples of R (M-R), denoted as M-R1, M-R3, and M-R7; pathogen-inoculated samples of R (In-R), denoted as In-R1, In-R3, and In-R7. In total, 24 samples were collected, and the test was repeated 2 times. The stems were rinsed with deionized water, and the samples were immediately frozen in liquid nitrogen and stored at − 80 ℃ for RNA extraction.

### Sample RNA extraction and detection

Total RNA from samples was extracted using the TRIzol Reagent kit (Invitrogen, ON, Canada). RNA degradation, contamination, and purity were monitored successively. Next, RNA concentration and integrity were measured. The mRNA was enriched from total RNA, PCR products were purified (AMPure XP system), and the library quality was assessed using the Agilent Bioanalyzer 2100 system.

The clustering of index-coded samples was performed on a cBot Cluster Generation System using TruSeq PE Cluster Kit v3-cBot-HS (Illumia, San Diego, USA). Reads of 50-bp length were generated using the Illumina HiSeq 2500 sequencing platform at Novogene Bioinformatics Technology Co. Ltd., Beijing, China.

### Sequencing, data processing, and reads mapping to the reference genome

Raw data (raw reads) were sequenced using Illumina HiSeq 2500. Reads containing adapter, containing ploy-N and low-quality reads from raw data were eliminated to obtain clean data (clean reads). Q30 and GC content of clean data were also calculated. Downstream analyzes were based on high-quality clean data.

The genome sequencing data of tobacco K326 on NCBI was used as a reference genome^25^. Clean reads were aligned to the reference genome by using TopHat v1.4.0 (http://ccb.jhu.edu/software/tophat/manual.shtml)^26,27^.

### Screening and cluster analysis of differentially expressed genes

Genes with an adjusted *p* value of < 0.05 were assigned as differentially expressed genes (DEGs) by differential expression analysis (DESeq R package, 1.10.1)^28^. Hierarchical clustering of all DEGs was performed using the R software (v 2.15.3) (https://cran.r-project.org/index.html), displayed by Heatmap.

### Gene ontology- and KEGG-enrichment analysis of DEGs

The GOseq R package was used to perform Gene Ontology (GO)-enrichment analysis of DEGs^29^. GO terms with a *p *value of < 0.05 were considered significantly enriched by DEGs. The Kyoto Encyclopedia of Genes and Genomes (KEGG) pathway-enrichment analysis^30^ was performed to identify significantly enriched metabolic or signal transduction pathways in tobacco DEGs. Pathways with a corrected *p *value of < 0.05 are considered significantly enriched in DEGs as assessed by the KOBAS software (KOBAS, Surrey, UK)^31^.

### Expression of qRT-PCR verification gene

Nine differentially regulated genes identified through DGE profiles were validated by qRT-PCR. HiScript Reverse Transcriptase (RNase H) (VAZYME, r101-01/02) was used to assist in cDNA synthesis starting from 1 μg of total RNA of resistant and susceptible cultivars at 3 and 7 dpi. Primers of selected genes were designed using the Primer Premier 5 software (PREMIER Biosoft, Palo Alto, CA, USA). Details of primer pairs are provided in Supplementary Table 1. SYBR Green Master Mix (VAZYME, Q111-02) was used for all qRT-PCR experiments in 20 μL of the reaction mix, comprising 2 × SYBR Green Master Mix (10 μL), cDNA (4 μL), PCR forward primer (100 μM; 0.4 μL), PCR reverse primer (100 μM; 0.4 μL), 50 × ROX reference dye 2^b^ (0.4 μL), and dd H_2_O (4.8 μL). The reaction procedure was performed on Applied Biosystems QuantStudio. Three replicates were performed with similar results. The *L25* gene was used as an internal reference for normalization^32^, and the relative gene-expression was calculated using the 2^−△△Ct^ method^33^.

### Pot experiment on controlling TBW by flavonoids

The *R. solanacearum* HBLC5 suspension (1 × 10^8^ CFU/mL) (OD600 = 0.1) was inoculated into wounds at the base of tobacco (Yunyan87, 5–6 leaves) stems under greenhouse conditions. At 12-h post-inoculation (hpi), flavonoids at concentrations of 0, 1, 2, and 4 mmol/L were poured into the base of tobacco stems (10 mL per plant, 8 seedlings per treatment, 3 replicates). The occurrence of TBW was recorded at 25 dpi, and the *I* and *DI* were calculated. The control efficiency (CE) was calculated using the following formula: CE (%) = (*DI—DI*_n_)/*DI*_0_ × 100%, n = 1, 2, and 4, where *DI*_0_ is the disease index of the treatment with 0 mmol/L flavonoid solution and *DI*_n_ is the disease index of the treatment with 1, 2, and 4 mmol/L of flavonoids, respectively.

## Results

### Disease symptoms of TBW in Yunyan87 and Fandi3

**Figure 1:**
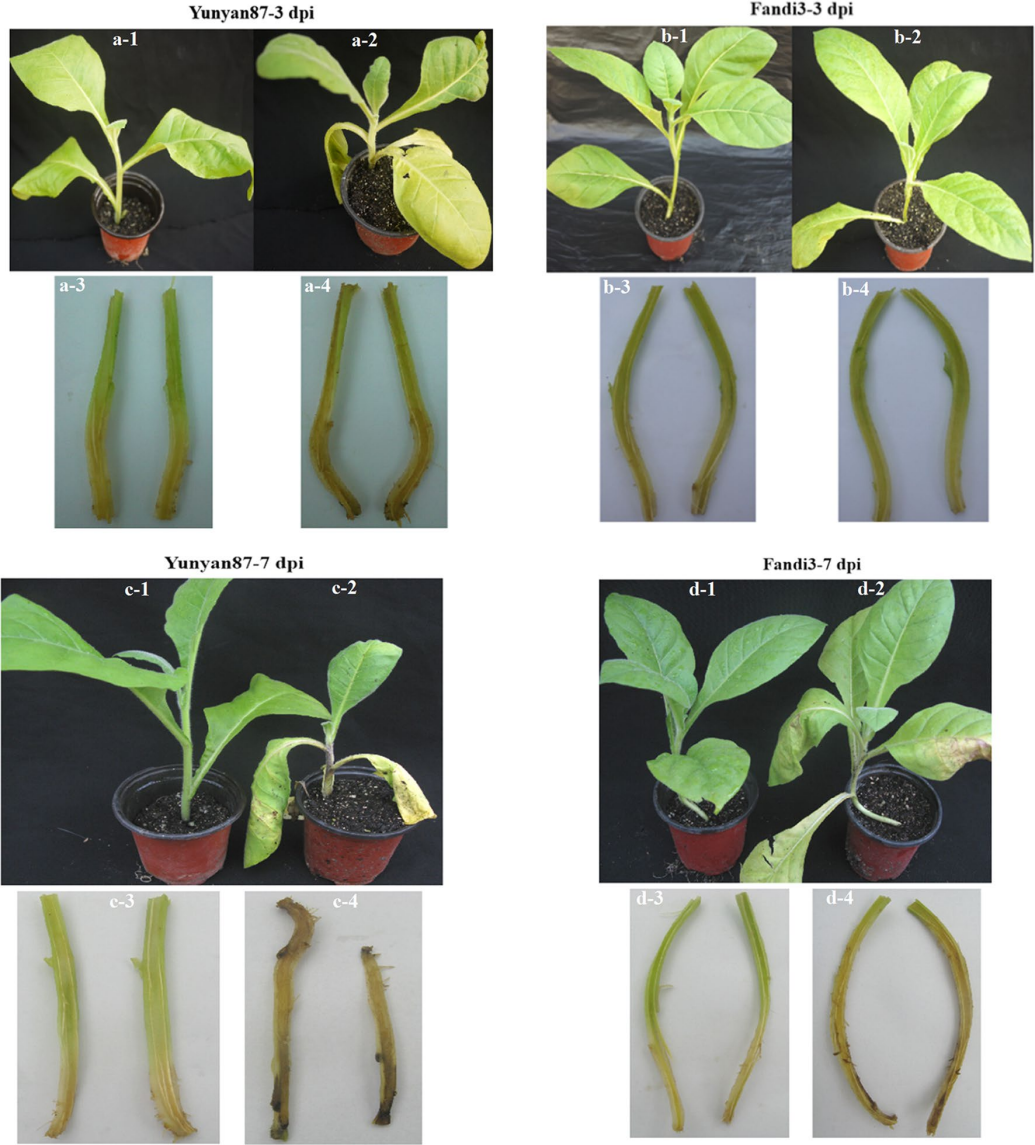
The disease symptoms of the resistant (Fandi3) and susceptible (Yunyan87) cultivars induced by *Ralstonia solanacearum* infection. Mock-inoculated seedling (**a-1**), *R. solanacearum-*inoculated seedling (**a-2**), mock-inoculated opened-stem (**a-3**), and *R. solanacearum-*inoculated opened-stem (**a-4**) of Yunyan87 at 3 dpi. Mock-inoculated seedling (**b-1**), *R. solanacearum-*inoculated seedling (**b-2**), mock-inoculated opened-stem (**b-3**), and *R. solanacearum-*inoculated opened-stem (**b-4**) of Fandi3 at 3 dpi. Mock-inoculated seedling (**c-1**), *R. solanacearum-*inoculated seedling (**c-2**), mock-inoculated opened-stem (**c-3**), and *R. solanacearum-*inoculated opened-stem (**c-4**) of Yunyan87 at 7 dpi. Mock-inoculated seedling (**d-1**), *R. solanacearum-*inoculated seedling (**d-2**), mock-inoculated opened-stem (**d-3**), and *R. solanacearum-*inoculated opened-stem (**d-4**) of Fandi3 at 7 dpi.

The resistance of Yunyan87 (susceptible cultivar) and Fandi3 (resistant cultivar) was determined by observing disease symptoms at 1, 3, 7, and 25 dpi and calculating *I* and *DI* (Table 1). At 3 dpi, TBW symptoms were observed in Yunyan87, with an *I* value of 9.09%, but not in Fandi3 (Fig. 1). At 7 dpi, *I* and *DI* values of Yunyan87 increased to 38.18% and 7.47, respectively, whereas TBW symptoms started to appear in Fandi3 (Fig. 1). At 25 dpi, *I* and *DI* values of Yunyan87 were 96.36% and 66.06, respectively, whereas the respective values increased to 34.55% and 12.73 in Fandi3. The findings indicated that Fandi3 was more resistant to TBW than Yunyan87.

**Table 1:** Disease incidence and index of tobacco seedlings inoculated with *R. solanacearum.*

Date of disease survey	Yunyan 87		Fandi 3	
	Incidence (%)	Disease index	Incidence (%)	Disease Index
1 dpi	0.00	0.00	0.00	0.00
3 dpi	9.09	1.01	0.00	0.00
7 dpi	38.18	7.47	7.27	1.21
25 dpi	96.36	66.06	34.55	12.73

### Illumina HiSeq sequencing

The RNA extracted from 24 samples were sequenced using the Illumina HiSeq 2500 platform. Overall, 299,673,192 raw reads, each 50 bp in length, were generated (Supplementary Table 2). After removing adaptor sequences, duplication sequences, ambiguous reads and low-quality reads, 296,721,446 high-quality clean reads (96.81–99.82% of the raw reads) were obtained. There were 93.36–97.13% of clean reads data from 24 samples at the Q30 level of Phred-like quality scores (an error probability of 0.001). About 94.55–96.98% of total clean reads were mapped uniquely to the reference genome. The findings demonstrated that sequenced data were reliable and suitable for further DGE analysis.

### Gene screening of differential expression

#### Venn analysis of DEGs

**Figure 2:**
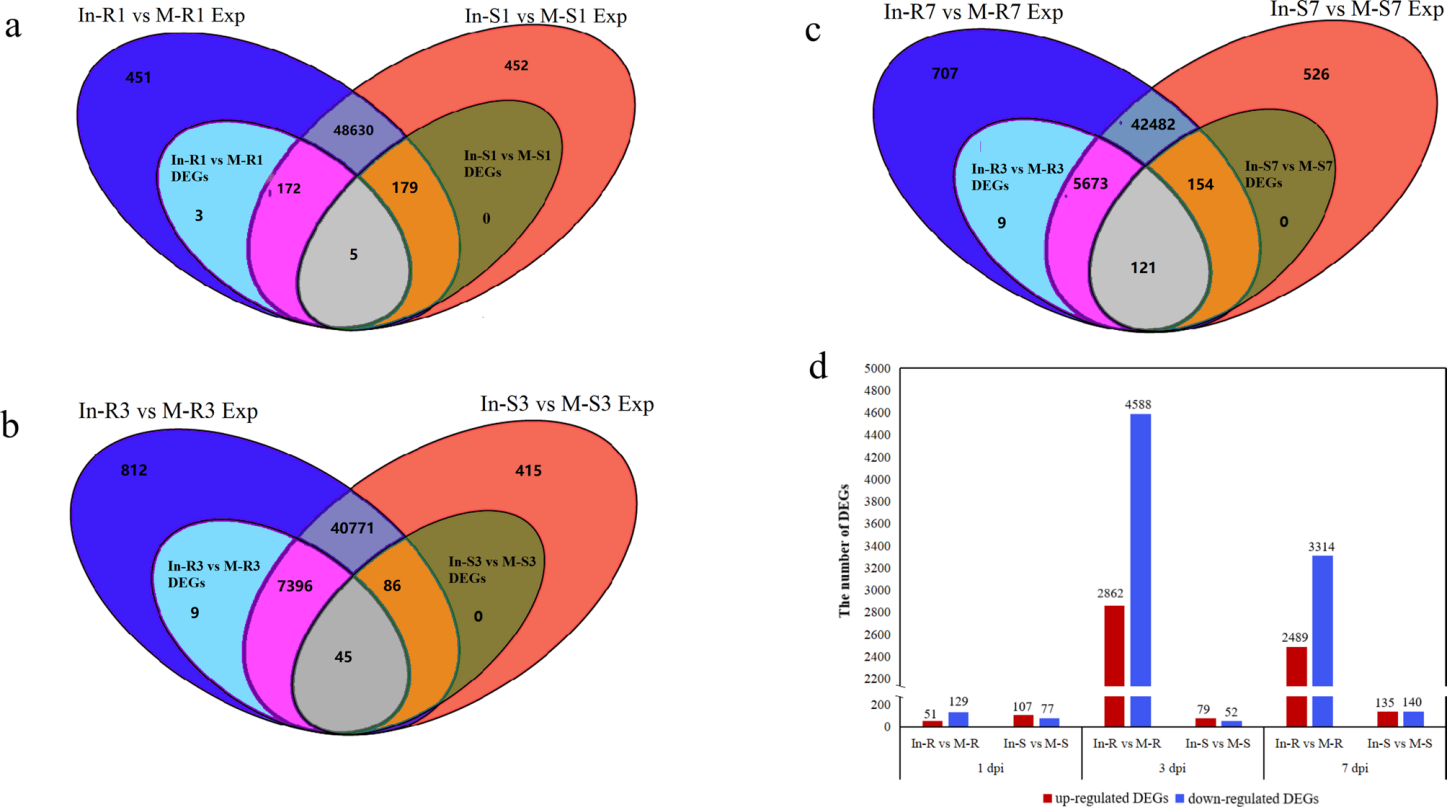
Differentially expressed genes (DEGs) in the resistant and susceptible cultivars inducing by *Ralstonia solanacearum* infection. Venn diagrams of all expressed genes and DEGs in resistant and susceptible cultivars at 1 (**a**), 3 (**b**), and 7 (**c**) dpi. In-R versus M-R Exp and In-R versus M-R DEGs indicated, compared with mock samples (M-R), the expressed genes and DEGs in the resistant cultivar inducing by *R. solanacearum* infection (In-R), respectively. In-S versus M-S Exp and In-S versus M-S DEGs indicated, compared with mock samples (M-S), the expressed genes and DEGs in the susceptible cultivar inducing by *R. solanacearum* infection (In-S), respectively. The number of upregulated and downregulated genes among DEGs in resistant and susceptible cultivars induced by *R. solanacearum* infection (**d**). Venn diagrams were generated in R (v 2.15.3) with the VennDiagram package (v 1.6.20, https://cran.r-project.org/web/packages/VennDiagram/index.html).

To identify potential genes involved in TBW resistance, all DEGs were identified in Fandi3 (R) and Yunyan87 (S) at 1, 3, and 7 dpi compared with mock-inoculated samples. The Venn diagram showed DEGs common to both cultivars or specific to either cultivar in response to inoculation (Fig. 2). At 1 dpi, 180 DEGs (*p* < 0.05, 129 downregulated and 51 upregulated) and 184 DEGs (*p* < 0.05, 77 downregulated and 107 upregulated) were identified in Fandi3 and Yunyan87, respectively (Fig. 2a, d). Of those DEGs, 3 were Fandi3-specific, 5 were common to both cultivars, 172 were DEGs (expressed but non-DEGs in Yunyan87) only in Fandi3, and 179 were DEGs (expressed but non-DEG in Fandi3) only in Yunyan87. At 3 dpi, 7450 DEGs (*p* < 0.05, 4588 downregulated and 2862 upregulated) and 131 DEGs (*p* < 0.05, 52 downregulated and 79 upregulated) were identified in Fandi3 and Yunyan87, respectively (Fig. 2b, d). Of those DEGs, 9 were Fandi3-specific, 45 were common to both cultivars, 7396 were DEGs only in Fandi3, and 86 were DEGs only in Yunyan87. At 7 dpi, 5803 DEGs (*p* < 0.05, 3314 downregulated and 2489 upregulated) and 275 DEGs (*p* < 0.05, 140 downregulated and 135 upregulated) were identified in Fandi3 and Yunyan87, respectively (Fig. 2c, d). Of those DEGs, 9 were Fandi3-specific, 121 were common to both cultivars, 5673 were DEGs only in Fandi3, and 154 were DEGs only in Yunyan87. The findings indicated that the number of DEGs in resistant cultivar (Fandi3) had considerably increased at 3 and 7 dpi, suggesting that these days may be important periods for the resistant cultivar's response to *R. solanacearum*.

#### Cluster analysis of DEGs

**Figure 3:**
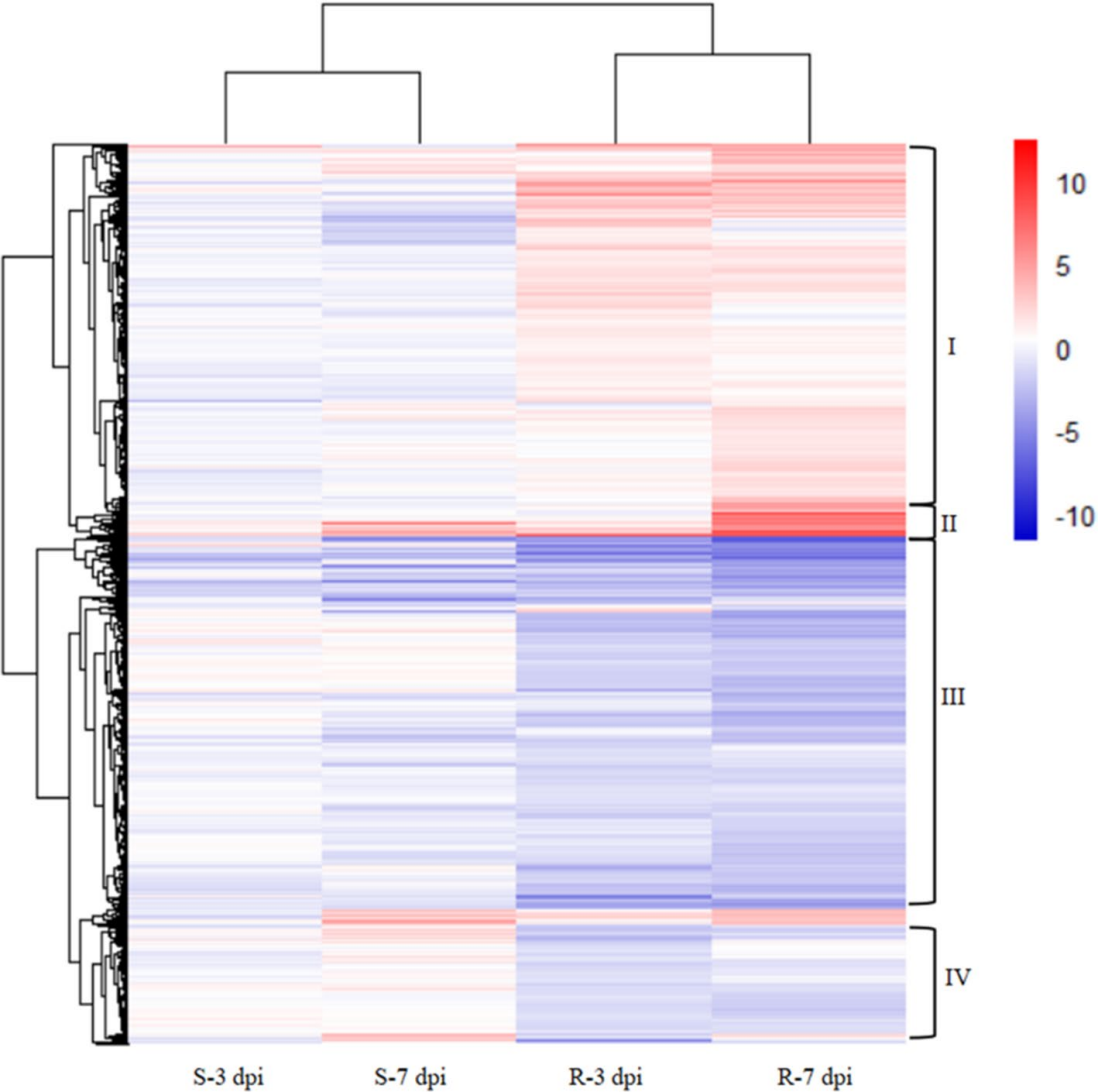
Expressions of differentially expressed genes in resistant and susceptible cultivars. Each column represents the log_2_ (In/M) at indicated times, where In and M are read count values of the gene in inoculated and mock samples, respectively. Red and blue represent upregulated and downregulated genes, respectively. The darker the color, the higher is the degree of upregulation or downregulation. Heatmap was generated in R (v 2.15.3) with the ComplexHeatmap package (v 2.11, https://bioconductor.org/packages/release/bioc/html/ComplexHeatmap.html).

A global view of expression profiles of tobacco’s resistance responses to *R. solanacearum* was obtained by classifying 9831 DEGs in both cultivars at 3 and 7 dpi using hierarchical clustering analysis. The heatmap revealed that these DEGs could be divided into 4 major groups (Fig. 3): genes upregulated in Fandi3 but downregulated in Yunyan87 (group I); genes upregulated in both cultivars (group II); genes downregulated in both cultivars (group III); and genes downregulated in Fandi3 but upregulated in Yunyan87 (group IV). It was speculated that genes in groups I and II, which were upregulated in Fandi3 but downregulated in Yunyan87 (group I) or upregulated in both cultivars (group II), could be involved in resistance responses to *R. solanacearum*.

### Analysis of DEGs by GO-enrichment

**Figure 4:**
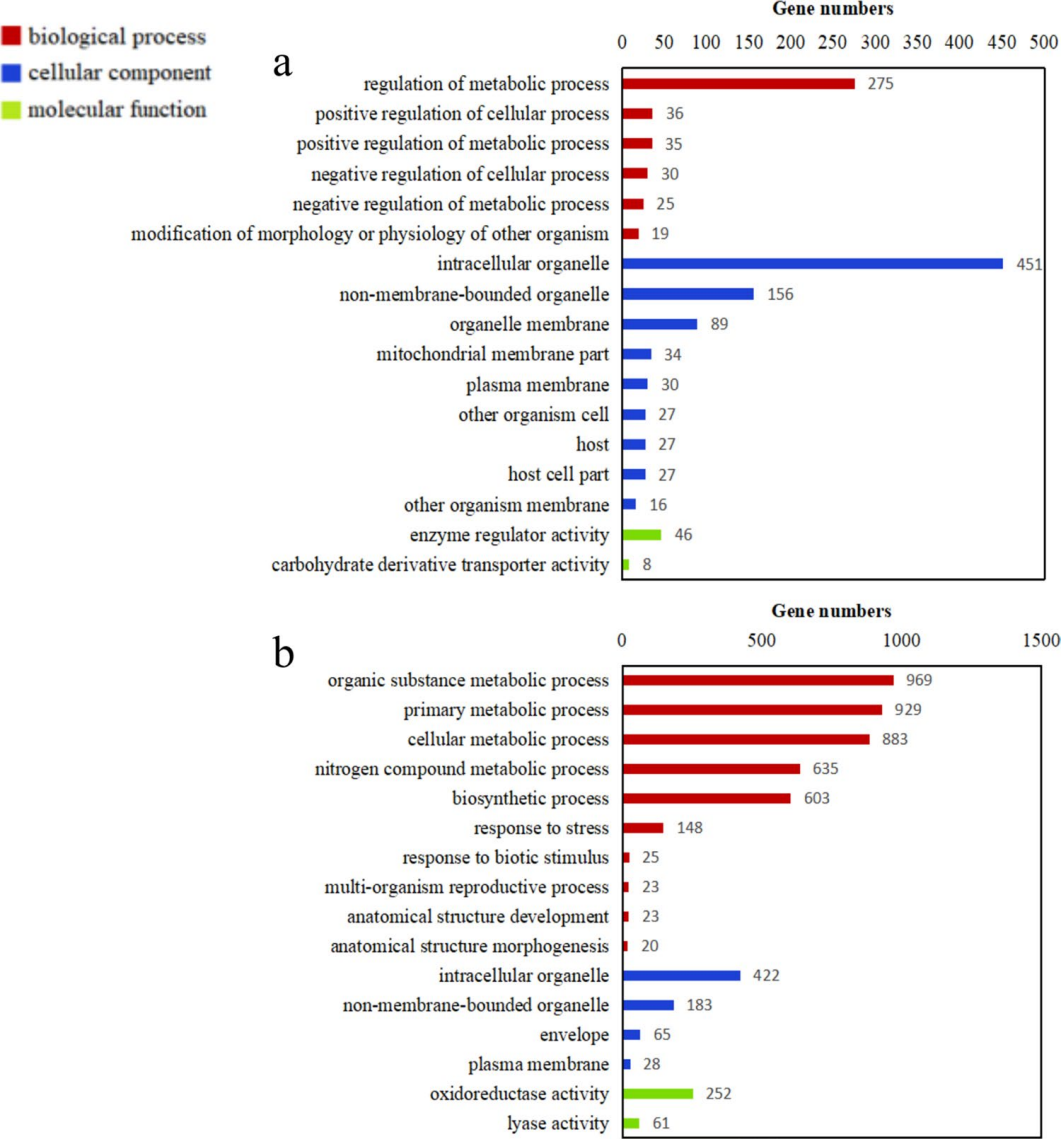
The upregulated Gene Ontology (GO) terms in the resistant cultivar induced by *Ralstonia solanacearum* infection. The upregulated GO terms in the resistant cultivar at 3 dpi (**a**) and 7 dpi (**b**).

To investigate biological processes and functions of DEGs possibly involved in tobacco’s resistance responses to *R. solanacearum*, we performed GO-enrichment analysis (molecular functions, biological processes, and cell components) to classify the functions of DEGs in groups I and II. At 3 dpi, 17 GO terms were significantly enriched (*p* ≤ 0.05), in which 2, 6, and 9 GO terms were classified as molecular functions, biological processes, and cell components, respectively (Fig. 4a). Of those, GO terms “intracellular organelle” and “regulation of metabolic process” were involved in most DEGs. At 7 dpi, 16 GO terms were significantly enriched (*p* ≤ 0.05), in which 2, 10, and 4 GO terms were classified as molecular functions, biological processes, and cell components, respectively (Fig. 4b). Of those, GO terms “organic substance metabolic process,” “primary metabolic process,” and “cellular metabolic process” were involved in more than 800 DEGs, respectively.

### KEGG annotation and pathway-enrichment analysis of DEGs

#### KEGG pathway analysis of DEGs

To further investigate the biosynthetic and signaling pathways of DEGs that could be involved in tobacco’s resistance responses to *R. solanacearum*, we performed pathway-enrichment analysis using KEGG. A total of 6 and 10 KEGG pathways (*p* ≤ 0.05) were involved in groups I and II DEGs (Table 2). These pathways were primarily involved in the phenylpropane pathway (phenylpropanoid biosynthesis, sly00940; flavonoid biosynthesis, sly00941; stilbenoid, diarylheptanoid, and gingerol biosynthesis, sly00945) and glutathione metabolism (sly00480)^34–36^.

**Table 2:** The up-regulated KEGG pathways in resistant cultivar inducing by R. solanacearum infection.

Pathway ID	Pathway	Gene number	corrected *P*-value
**3 dpi**			
sly03040	Spliceosome	55	1.135E−06
sly00945	Stilbenoid, diarylheptanoid and gingerol biosynthesis	17	3.274E−04
sly00941	Flavonoid biosynthesis	17	0.003
sly00960	Tropane, piperidine and pyridine alkaloid biosynthesis	13	0.016
sly03010	Ribosome	66	0.017
sly00940	Phenylpropanoid biosynthesis	38	0.040
**7 dpi**			
sly03010	Ribosome	138	3.228E−17
sly00480	Glutathione metabolism	38	1.400E−04
sly00945	Stilbenoid, diarylheptanoid and gingerol biosynthesis	17	0.004
sly00400	Phenylalanine, tyrosine and tryptophan biosynthesis	20	0.008
sly00941	Flavonoid biosynthesis	18	0.010
sly00940	Phenylpropanoid biosynthesis	45	0.010
sly00270	Cysteine and methionine metabolism	29	0.010
sly00360	Phenylalanine metabolism	41	0.010
sly04141	Protein processing in endoplasmic reticulum	57	0.011
sly01230	Biosynthesis of amino acids	55	0.021

#### Differential expression analysis of genes related to the KEGG pathway

**Figure 5:**
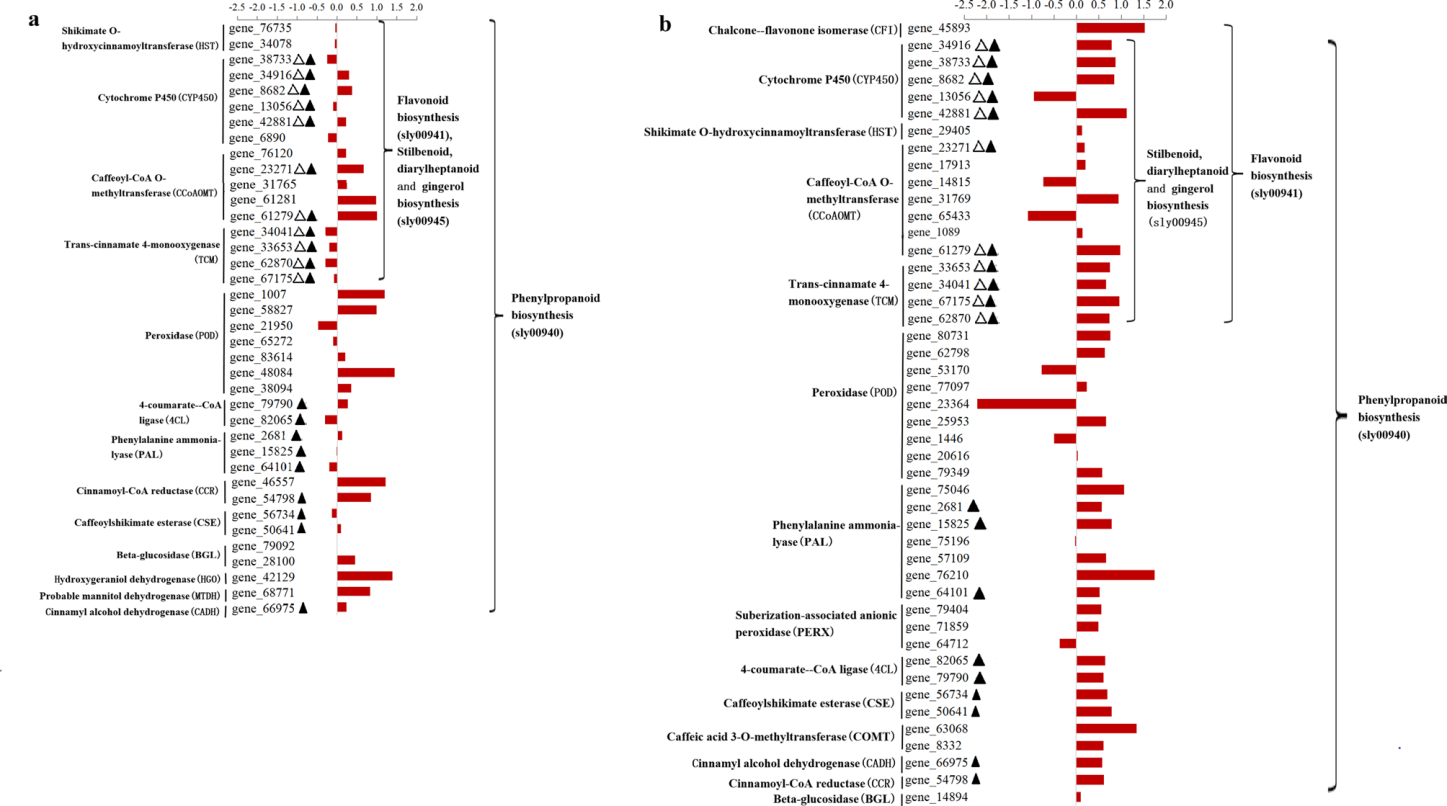
Comparison of the expression of genes associated with the phenylpropane pathway in resistant and susceptible cultivars in response to *Ralstonia solanacearum* infection. The bar represents log_2_ (In-R/In-S), where In-R and In-S are read count values of the gene in resistant and susceptible cultivars induced by *R. solanacearum*. Comparison of the expression of genes in resistant and susceptible cultivars in response to *R. solanacearum* infection at 3 dpi (**a**) and 7 dpi (**b**). Δ indicated common differentially expressed genes enriched in the flavonoid biosynthesis and stilbenoid, diarylheptanoid, and gingerol biosynthesis pathway at 3 and 7 dpi. Filled triangle indicated common differentially expressed genes enriched in the phenylpropanoid biosynthesis pathway at 3 and 7 dpi.

Phenylalanine is a precursor of several secondary metabolites in plants. In the phenylpropanoid biosynthesis pathway, phenylalanine is transformed into p-coumaroyl CoA by phenylalanine ammonia-lyase (PAL) and 4-coumaric acid CoA ligase (4CL). Finally, through a series of biochemical reactions, p-coumaroyl CoA is converted to flavonoids, stilbenoids, and lignins (Supplementary Figs. 1–3). Our results demonstrated that 38 upregulated DEGs (0.79–5.38 folds) in Fandi3 were enriched in the phenylpropane pathway at 3 dpi, in which the expression levels of 23 genes (23/38, 60.53%) were higher in Fandi3 than in Yunyan87. At 7 dpi, 46 upregulated DEGs (1.49–8.92 folds) in Fandi3 were enriched in the phenylpropane pathway, in which the expression levels of 38 genes (38/46, 82.61%) were higher in Fandi3 than in Yunyan87 (Fig. 5, Supplementary Table 3). Moreover, 20 upregulated DEGs in Fandi3 involved in the phenylpropane pathway were identified at 3 and 7 dpi. They were related to PAL, 4CL, trans-cinnamate 4-monooxygenase (TCM), cytochrome P450 (CYP450), caffeoyl-CoA O-methyltransferase (CCoAOMT), cinnamoyl-CoA reductase (CCR), caffeoylshikimate esterase (CSE), and cinnamyl alcohol dehydrogenase (CADH). The upregulated DEGs may increase the accumulation of flavonoids, stilbenoids, and lignins, enhancing the plant’s resistance against *R. solanacearum*.

**Figure 6:**
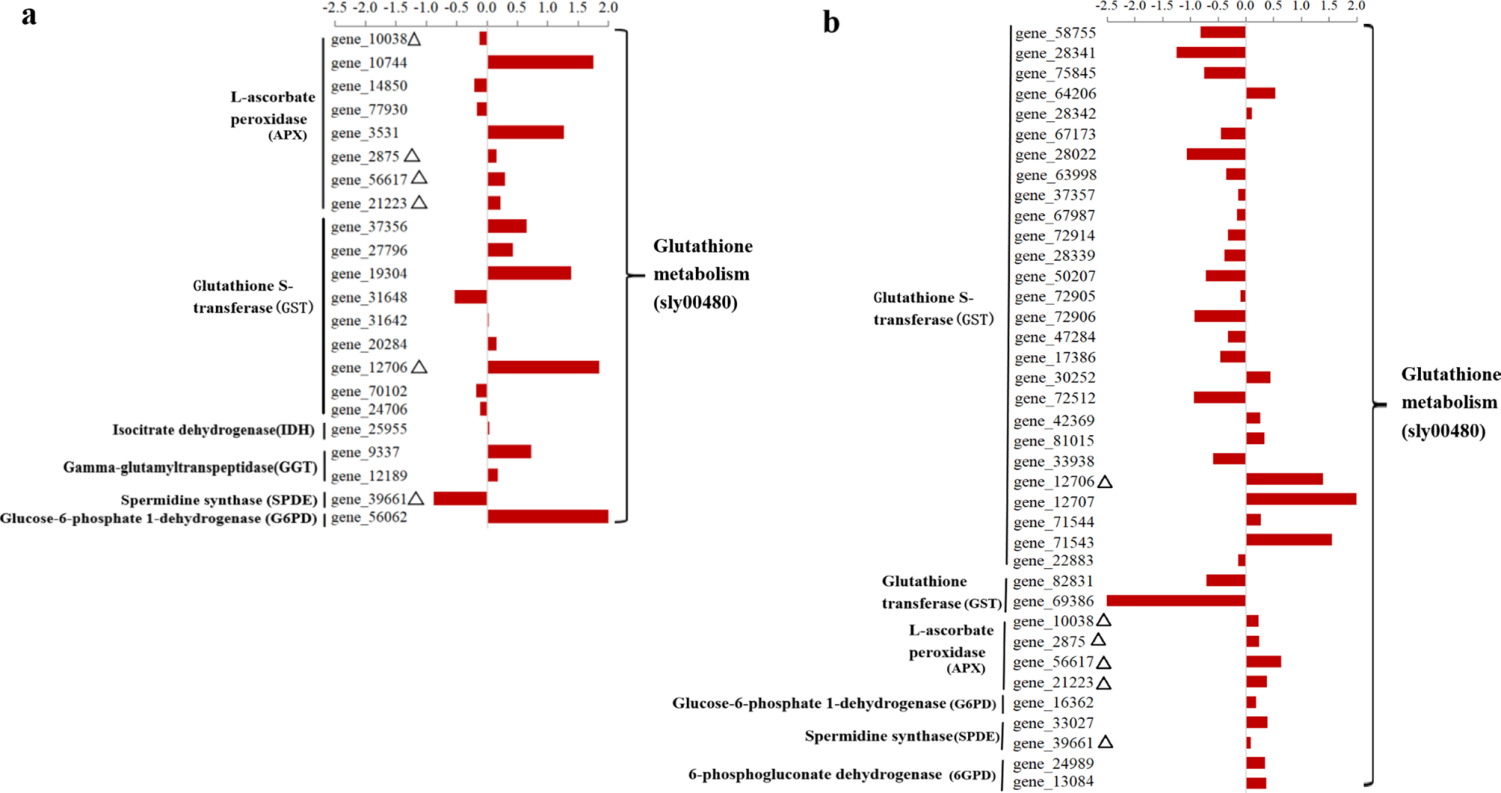
Comparison of the expression of genes associated with glutathione metabolism in resistant and susceptible cultivars in response to *Ralstonia solanacearum* infection. The bar represents log_2_ (In-R/In-S), where In-R and In-S are read count values of the gene in resistant and susceptible cultivars induced by *R. solanacearum*. Comparison of the expression of genes in resistant and susceptible cultivars in response to *R. solanacearum infection* at 3 dpi (**a**) and 7 dpi (**b**). Δ indicated common differentially expressed genes enriched in the glutathione metabolism at 3 and 7 dpi.

In the glutathione metabolic pathway (Supplementary Fig. 4), 22 (0.77–6.02 folds) and 38 (1.76–10.50 folds) upregulated DEGs in Fandi3 were enriched at 3 and 7 dpi, respectively (Fig. 6, Supplementary Table 4). Moreover, 6 upregulated DEGs involved in the glutathione metabolic pathway were identified at both 3 and 7 dpi, and they were associated with L-ascorbate peroxidase (APX), glutathione S-transferase (GST), and spermidine synthase (SPDE).

### Transcription factor and PR-related gene-expression

WRKY TFs, ERFs, and pathogenesis-related proteins (PRs) play key roles in plant resistance against pathogens, implying the importance of investigating them at 3 and 7 dpi in this study.

**Figure 7:**
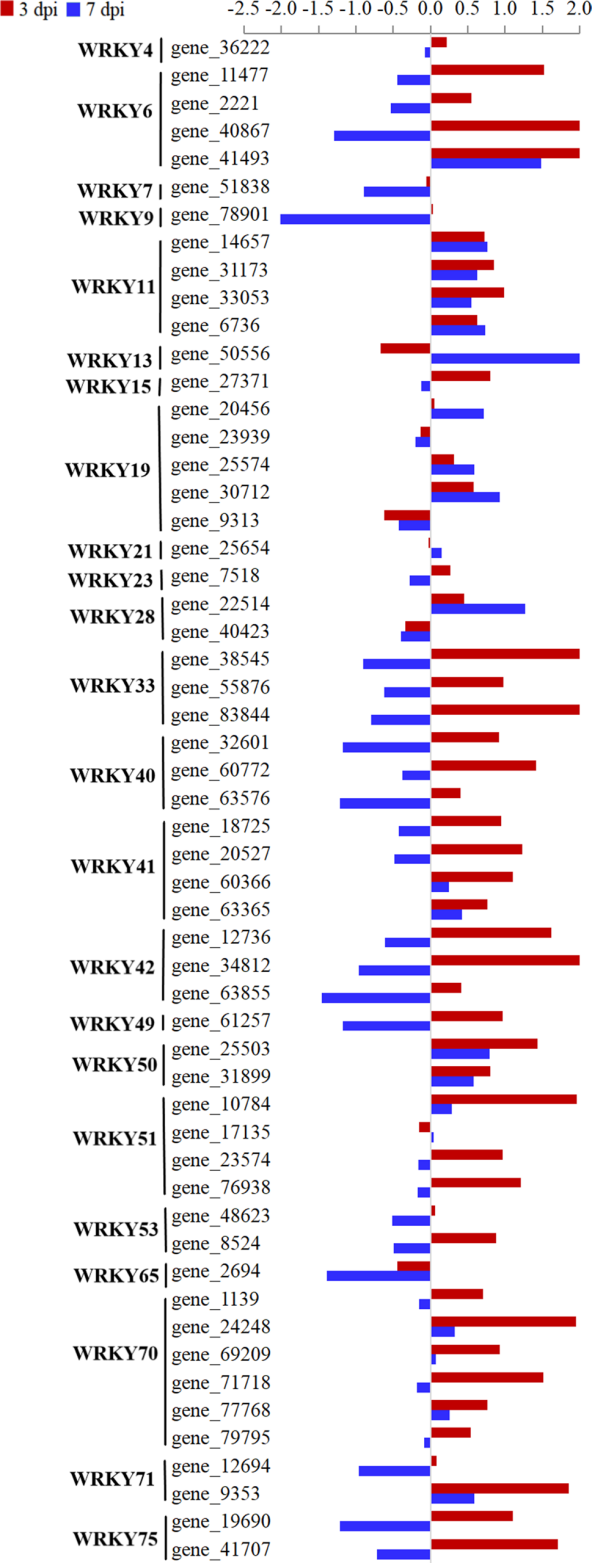
Comparison of the expression of genes associated with WRKY transcription factors in resistant and susceptible cultivars in response to *Ralstonia solanacearum* infection. The bar represents log_2_ (In-R/In-S), where In-R and In-S are read count values of the gene in resistant and susceptible cultivars induced by *R. solanacearum*.

WRKY TFs are the largest family of proteins in plants that regulate a series of defense processes and play important roles in modulating the transcription of resistance-related genes. In this study, the expressions of 55 WRKY genes were identified in both cultivars (Fig. 7, Supplementary Table 5). Of these, 15 WRKY genes (WRKY6, WRKY11, WRKY23, WRKY28, WRKY33, WRKY41, WRKY49, and WRKY65 family genes) were significantly upregulated in Fandi3 at 3 dpi, and 18 WRKY genes (WRKY6, WRKY11, WRKY15, WRKY21, WRKY40, WRKY41, WRKY50, WRKY51, WRKY70, and WRKY75 family genes) were significantly upregulated in Fandi3 at 7 dpi. Notably, 3 genes, *gene_14657*, *gene_31173*, *and gene_6736* (WRKY11 family genes), were significantly upregulated in Fandi3 at both 3 and 7 dpi, and their expression levels were higher in Fandi3 than in Yunyan87. It is speculated that genes involved in WRKY11 may play an important role in tobacco resistance against *R. solanacearum.* Moreover, the expression levels of genes in the WRKY6 family (except *gene_41493*) could be important positive regulators of resistance against *R. solanacearum* in pepper^13^ identified only at 3 dpi to be higher in Fandi3 than in Yunyan87. This indicated that WRKY6 family genes might respond to *R. solanacearum* infection, especially in the early stage.

**Figure 8:**
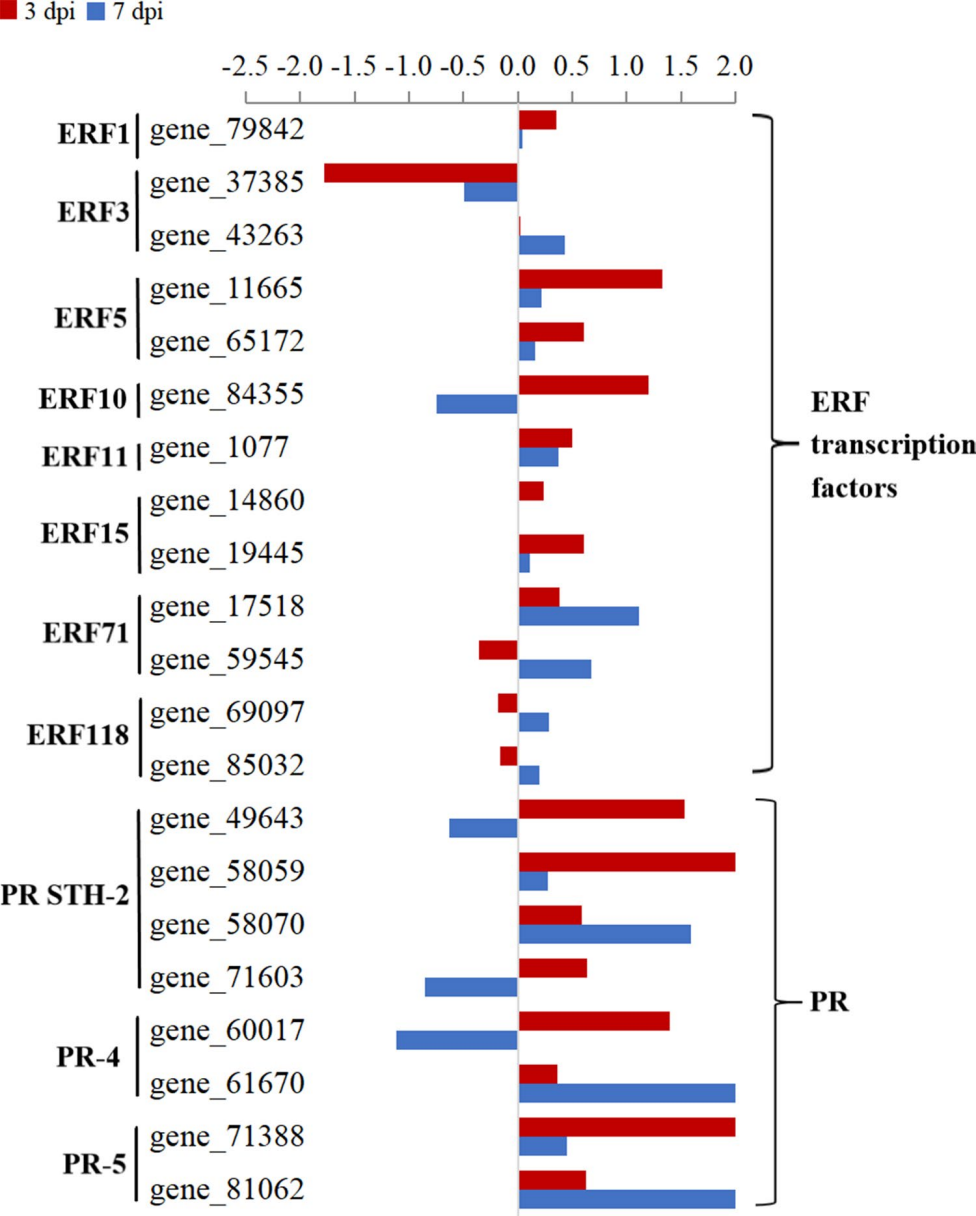
Comparison of the expression of genes associated with ERF transcription factors and pathogenesis-related proteins (PR) in resistant and susceptible cultivars in response to *Ralstonia solanacearum* infection. The bar represents log_2_ (In-R/In-S), where In-R and In-S are read count values of the genes in resistant and susceptible cultivars induced by *R. solanacearum.*

ERFs have important biological functions in various life activities such as plant growth, development, and response to the environment. In this study, the expression of 13 ERFs genes was identified in both cultivars (Fig. 8, Supplementary Table 5). Of those, in Fandi3, 5 ERF genes (ERF3, ERF5, ERF10, and ERF15 family genes) were significantly upregulated at 3 dpi, and 5 (ERF10, ERF15, and ERF71 family genes) were significantly upregulated at 7 dpi. Moreover, *gene_14860* and *gene_19445* (ERF15 family genes) were significantly upregulated in Fandi3 at both 3 and 7 dpi, and their expression levels were higher than that in Yunyan87. These findings suggested that these 2 genes could be important positive regulators of tobacco resistance against *R. solanacearum*. Further, the expression level of *gene_11665* in the ERF5 family, which could upregulate the expression levels of a series of defense-related genes in plants^22^, was identified only at 3 dpi and was higher in Fandi3 than in Yunyan87. This result implied that this gene might also respond to *R. solanacearum* infection primarily in the early stage.

PRs are induced by phytopathogens and defense-related signaling molecules. They often serve as diagnostic molecular markers of defense signaling pathways. PRs are effective against multiple biotic agents such as fungi, bacteria, or even insects^37,38^. Overall, 8 PR genes were identified in both cultivars (Fig. 8, Supplementary Table 5). In Fandi3, one PR gene was significantly upregulated at 3 dpi, and 4 PR genes were significantly upregulated at 7 dpi. Of those, genes encoding PR5 were significantly upregulated in Fandi3, and the expression levels of the genes were higher than that in Yunyan87. The findings suggested that PR5 could be a key positive regulator in tobacco’s defense against *R. solanacearum* infection.

### Expression verification of genes associated with resistance against TBW

**Figure 9:**
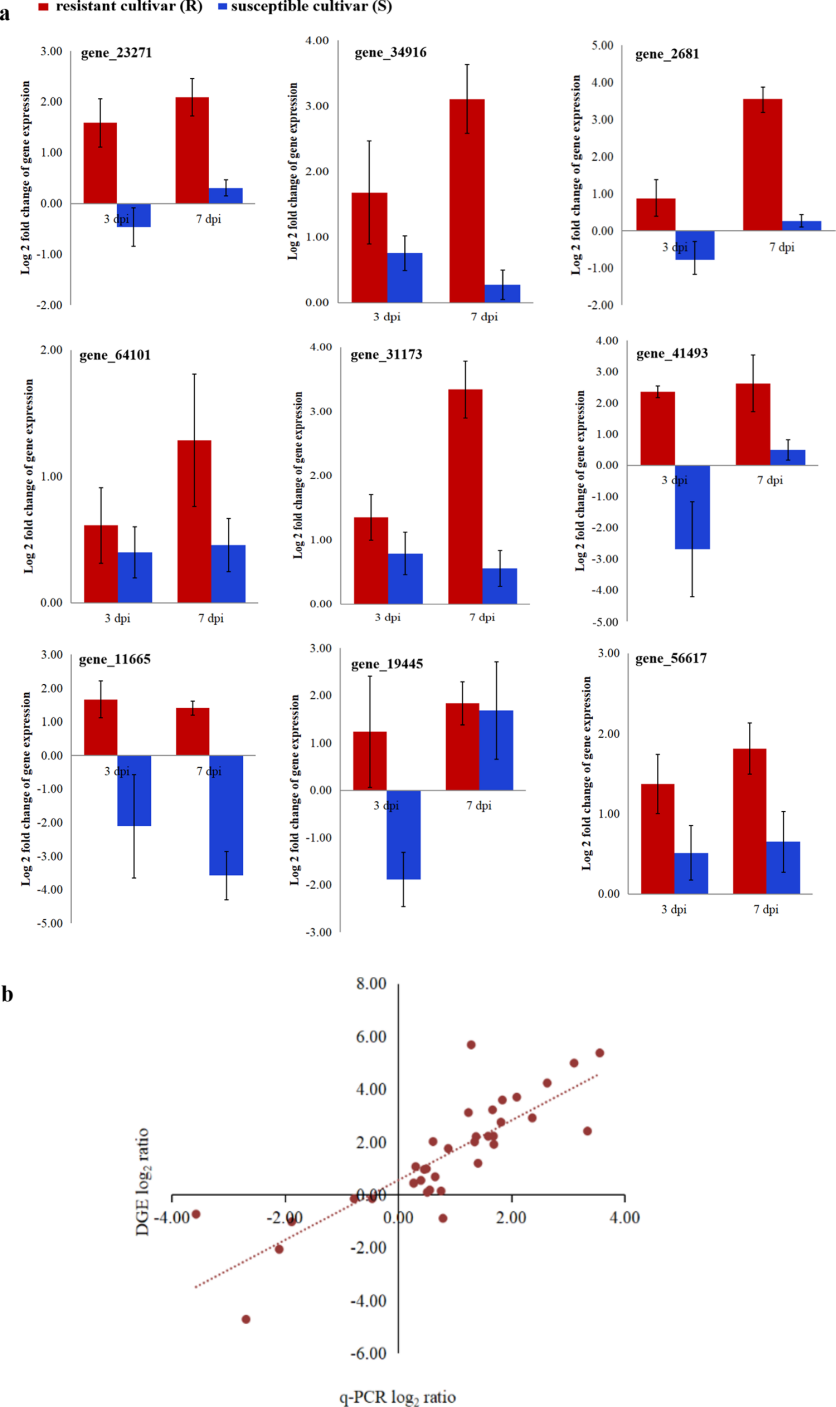
Verification of the digital gene expression (DGE) profile. Expression levels of the candidate differentially expressed genes obtained by qRT-PCR, calculated using the 2^−△△Ct^ method with normalization to the internal reference *L25* gene (**a**). The linear correlation between DGE profiles and qRT-PCR data (**b**). A correlation coefficient of 0.835 indicates an excellent linear correlation between the DGE profile and qRT-PCR data.

Nine DEGs that could be essential for resistance were selected to determine the relative expression level by qRT-PCR at 3 and 7 dpi. The genes were one gene (*gene_34916*) associated with CYP450, one gene (*gene_23271*) associated with CCoAOMT, 2 genes (*gene_2681* and *gene_64101*) associated with PAL, one gene (*gene_56617*) associated with APX, 2 genes (*gene_41493* and *gene_31173*) associated with WRKY TFs, and 2 genes (*gene_11665* and *gene_19445*) associated with ERFs. The results revealed that all 9 genes were upregulated at 3 and 7 dpi in Fandi3 but had lower expression levels or downregulated in Yunyan87 (Fig. 9a). The correlation coefficient between DGE and qRT-PCR data was significant at 0.835 (*p* < 0.01), indicating that qRT-PCR data were consistent with DGE data and confirmed expression patterns of these genes as revealed by DGE data (Fig. 9b).

### Control efficiency of flavonoids on TBW

KEGG-enrichment analysis of DEGs demonstrated that genes related to plant protectin synthesis in the phenylpropane pathway were significantly upregulated in Fandi3. To verify whether flavonoids were involved in resistance against TBW, flavonoids at various concentrations were applied on the stem base of Yunyan87 inoculated with 1 × 10^8^ CFU/mL of *R. solanacearum* under greenhouse conditions. The *DI* of tobacco seedlings treated with 1, 2, and 4 mmol/L flavonoids were significantly lower than those treated with 0 mmol/L flavonoids at 25 dpi. The control efficiency values of flavonoids at 1, 2, and 4 mmol/L on bacterial wilt were 56.10%, 59.76%, and 84.15%, respectively (Table 3).

**Table 3:** The control efficiency of flavonoids on tobacco bacterial wilt.

The concentration of flavonoid (mmol/L)	Incidence (%)	Disease Index	Control efficiency (%)
1	33.33 ± 0.00 b	22.22 ± 0.00 b	56.10
2	27.78 ± 9.62 b	20.37 ± 12.96 b	59.76
4	16.67 ± 0.00 b	8.02 ± 2.14 b	84.15
0	66.67 ± 16.67 a	50.62 ± 11.16 a	

Different lowercase letters indicated that the disease incidence and disease index of TBW showed significant differences at *P* < 0.05 among different treatments.

## Discussion

To study the mechanism of the tobacco plant’s resistance against TBW, DEGs at different times were analyzed after inoculation with *R. solanacearum*. At 1 dpi, the number of DEGs in both cultivars was fewer, indicating that the resistance response to TBW was not activated. However, resistant-related genes were quickly activated in Fandi3, with the number of upregulated genes being 36.23 and 18.44 folds higher in Fandi3 than in Yunyan87 at 3 and 7 dpi, respectively. This finding is consistent with previous studies^7,39^ and demonstrated that Fandi3 (resistant cultivar) had a significant resistance response to TBW at 3 and 7 dpi.

Certain metabolic pathways play essential roles in plant resistance against TBW. Glutathione (GSH) is the most abundant antioxidant in cells and is crucial for life processes by protecting DNA, proteins, and other biomolecules against oxidative damage and heavy metal ions, which is favorable to the resistance against environmental stress^40–42^. Avinash et al.^43^ demonstrated that the expression of resistance genes associated with the ascorbic acid (AsA)-GSH pathway increased by 2.5–3.5 folds in the resistant eggplant cultivars after infection with *R. solanacearum*. In our study, after infection with *R. solanacearum*, the expression of genes associated with the GSH metabolism pathway increased significantly in Fandi3. The genes associated with APX increased by 2.16 and 3.61 folds at 3 and 7 dpi, respectively, and genes associated with GST increased by 4.15 and 4.01 folds at 3 and 7 dpi, respectively. APX is the first step of the AsA-GSH cycle and is an effective antioxidant enzyme that assists in removing H_2_O_2_ from cells^44^. GST is an important antioxidant enzyme related to GSH metabolism and controls the binding of GSH to certain biomolecules^45^. Therefore, the upregulated genes associated with APX and GST in plants could enhance the redox ability and protect cell membranes from damage caused by reactive oxygen species (ROS).

The phenylpropane pathway plays an essential role in plant disease-resistance. In the present study, genes associated with PAL and 4CL were significantly upregulated in Fandi3, which is in line with the study by Ishihara et al.^6^. They demonstrated that genes associated with 4CL were significantly upregulated in resistant tomato cultivars after infection with *R. solanacearum*, indicating that the infection could lead to the accumulation of phenylpropane derivatives in resistant cultivars. There are 2 main branching pathways in the phenylpropanoid biosynthesis pathway: the lignin pathway and the phenols and flavonoids pathway. Lignin is an important component of the plant secondary cell wall. Certain enzymes such as CADH, CSE, and CCoAOMT play central roles in lignin synthesis^46,47^. In the present study, genes associated with CADH, CSE, and CCoAOMT were significantly upregulated in Fandi3 after infection, consistent with the response of resistant tomato cultivars after infection with *R. solanacearum*^6^. This could be because bacterial wilt could induce lignin accumulation in plants as a physical barrier for resistance against the infection. Flavonoids and astragalus compounds as protective agents can inhibit several pathogenic bacteria^48^. In this study, the expression of genes associated with the biosynthesis of flavonoids, stilbenes, diarylheptanoids, and gingerol increased significantly in Fandi3, indicating that the infection of bacterial wilt in resistant variety could promote the accumulation of flavonoids and stilbenes to inhibit the reproduction of *R. solanacearum* and improve resistance against the infection. This finding is consistent with the conclusion of Chen et al. that bacterial wilt disease promoted the biosynthesis of flavonoids and terpenoids in resistant peanut lines^49^. CYP450 is an essential metabolic enzyme present in most organisms. It is mainly involved in metabolic pathways of hormones, phenylpropane, alkaloids, and terpenes^50–53^. In this study, genes associated with CYP450 in the phenylpropane pathway were significantly upregulated in Fandi3, indicating that CYP450 could be mainly involved in the accumulation of phenylpropane derivatives to improve the resistance of tobacco to bacterial wilt.

Yang et al. demonstrated that rice, tobacco, and *Arabidopsis thaliana* pretreated with flavonoids had enhanced resistance to *Xanthomonas oryzae pv. oryzae*, *R. solanacearum*, and *Pseudomonas syringae pv. tomato*, respectively^54^. Moreover, certain PR-related genes such as *PR1a*, *NOA1*, and *rbohB* were significantly upregulated in tobacco leaves treated with flavonoids. In this study, KEGG-enrichment analysis of DEGs revealed that genes related to plant protectin synthesis in the phenylpropane pathway were significantly upregulated in Fandi3, indicating the possible involvement of flavonoids in resistance against TBW. We, therefore, performed a pot experiment to evaluate the role of flavonoids in controlling TWB. The control efficiency of 1–4 mmol/L flavonoids on controlling TBW was 56.10–84.15%. As such, the reasons for the exogenous application of flavonoids improving resistance against TBW could be flavonoid-induced upregulation of PR genes, but this aspect requires further evidence.

TFs are the most important regulatory genes in plants. WRKY TFs play essential roles in the immune response of plants to various biological stresses^55^. Many studies have shown that *WRKY40*, *WRKY6*, *WRKY27*, and *WRKY22* have positive regulatory effects on the resistance of *Solanaceae* crops to bacterial wilt^14–18^], and *CaWRKY6* regulated *CaWRKY40* to coordinate the response to bacterial wilt^15^. In this study, WRKY6 had a resistance response to TBW, mainly in the early stage (3 dpi), which is consistent with the response pattern of pepper to bacterial wilt^15^. Moreover, WRKY11 family genes demonstrated apparent positive regulation in Fandi3 and negative regulation in Yunyan87, suggesting these genes could also be important positive regulators of tobacco resistance against *R. solanacearum*^56,57^, which needs further verification. ERFs are a unique class of TFs in plants involved in response to biological and abiotic stresses^58,59^. Zhang et al.^60^ reported that the expression of *ERF15* could be induced by *P. syringae* pv. *tomato* and *Botrytis cinerea* infection and *ERF15* overexpression significantly increased resistance in plants. In this study, the expression of ERF15 family genes was significantly upregulated (3.74 and 3.58 folds at 3 and 7 dpi, respectively) in Fandi3 after infection, indicating that the gene is an important positive regulator of tobacco resistance against *R. solanacearum*. The expression pattern of *ERF5* (*gene_11665*) in Fandi3 was distinct from that in Yunyan87, especially in the early stage (3 dpi). It could have regulated a series of defense genes to enhance the resistance of tobacco^22^. PRs are generally considered important for the systemic acquisition of disease-resistance (SAR)^61^. Dahal et al.^62^ pointed out that PR5 had a positive regulatory effect on tomato resistance against bacterial wilt. In this study, PR5-related genes demonstrated significant regulatory differences between Fandi3 and Yunyan87 after infection, in which the genes in Fandi3 were upregulated by 1.31 and 2.38 folds on average at 3 and 7 dpi after infection, respectively, whereas genes in Yunyan87 were downregulated. The results indicated the possible involvement of PR5 in tobacco resistance against *R. solanacearum*.

The 9 genes in Fandi3 and Yunyan87 were further analyzed using qRT-PCR. The genes were either upregulated in Fandi3 at both 3 and 7 dpi or demonstrated a distinct expression pattern in Fandi3 and Yunyan87 in DGE. Further, these genes involved in key pathways (phenylpropane pathway and glutathione metabolism) were related to PAL, CYP450, CCoAOMT, and APX or were WRKY genes and ERFs genes. Therefore, it was necessary to select these genes for verifying the DEGs identified and confirmed the expression patterns of these genes in Fandi3 and Yunyan87 by qRT-PCR.

## Conclusions

In this study, the DGE sequencing technology was used to analyze DEGs and related pathways in resistant and susceptible tobacco cultivars. Both cultivars had similar transcriptome levels at the early stage of infection, but resistance-related DEGs had distinct genotype-specific expression patterns in response to TBW after 3 dpi. Meanwhile, glutathione metabolism and phenylpropane pathway were the main resistant pathways of tobacco’s response to *R. solanacearum*. Furthermore, a pot experiment suggested that flavonoids help control TWB. The study results reveal the molecular mechanisms involved in tobacco resistance to *R. solanacearum* and serve as an important guide for selecting and breeding tobacco plants resistant to TBW.

## Supplementary Information

Supplementary Figures

Supplementary Tables
