# Supplementary material for: Digital gene expression analysis of the response to Ralstonia solanacearum between resistant and susceptible tobacco varieties
Source: Sci Rep. 2021 Feb 16;11:3887. doi: 10.1038/s41598-021-82576-8 (PMC7886896; doi:10.1038/s41598-021-82576-8)
Supplement: Supplementary file 1 — Supplementary Figures [file 41598_2021_82576_MOESM1_ESM.pdf]

Digital gene expression analysis of the response to *Ralstonia solanacearum* between resistant and susceptible tobacco varieties

YanYan Li[1#], Lin Wang[2#], GuangWei Sun[1], XiHong Li[1], ZhenGuo Chen[1], Ji Feng[1*] and Yong Yang[3*]

[1] Tobacco Research Institute of Hubei Province, Wuhan, 430030, China;

[2] China Tobacco Hubei Industrial Co., Ltd., Wuhan, 430040, China.

[3] School of life sciences, Hubei University, Wuhan, 430062, China;

# Yanyan Li and Lin Wang contributed equally to this work.

*Corresponding author: Associate Professor Yong Yang and Ph.D Ji Feng, E-mail addresses: yangyong@hubu.edu.cn, fengji1981@163.com

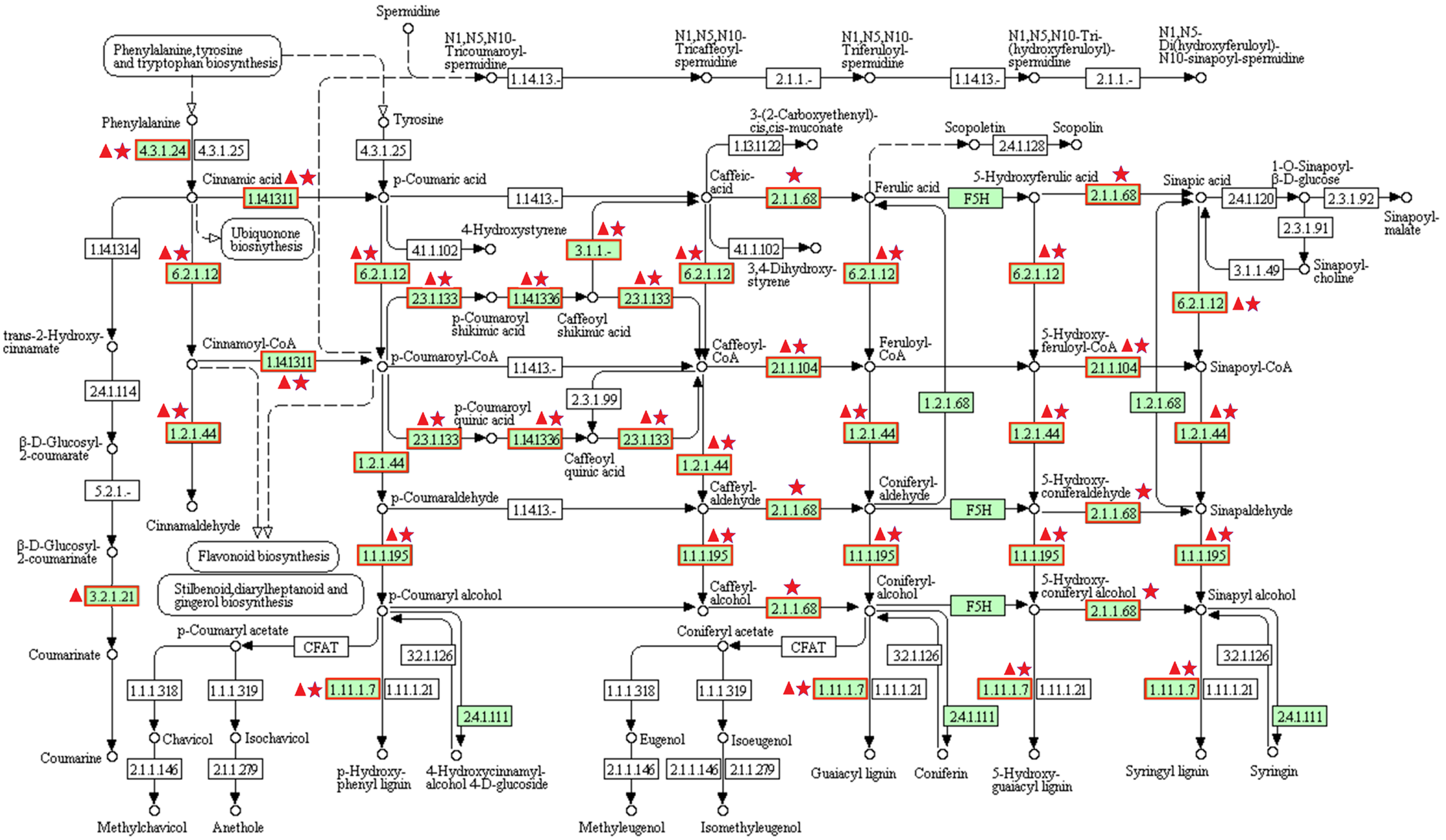

Supplementary Fig.S1. Phenylpropanoid biosynthesis pathway (sly00940) and the differentially expressed genes related the pathway. The red box indicated the up-regulated differentially expressed genes related to phenylpropanoid biosynthesis pathway in resistant cultivars response to *R. solanacearum* at 3 dpi (▲) and 7 dpi (★), respectively.

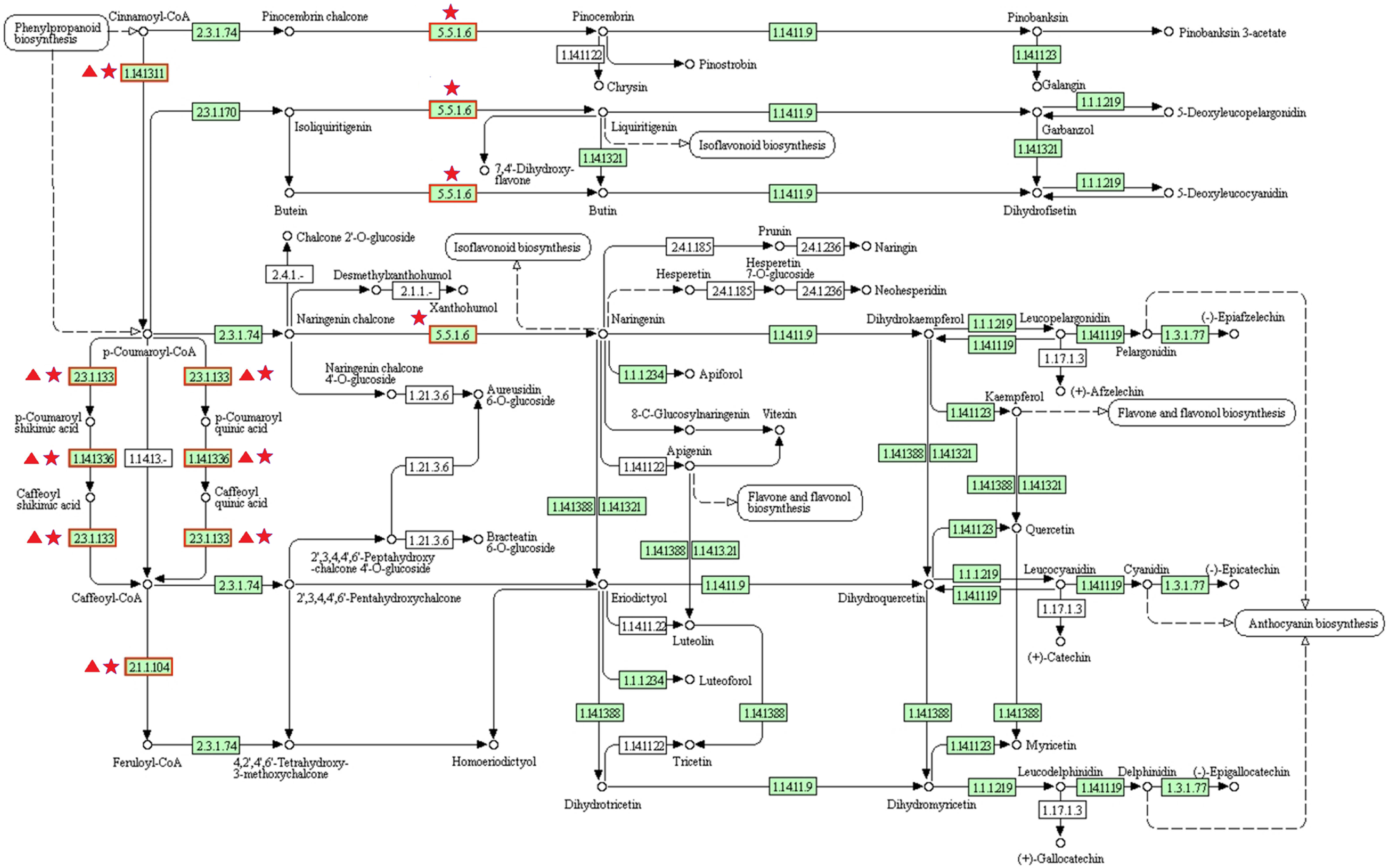

Supplementary Fig. S2. Flavonoid biosynthesis pathway (sly00941) and the differentially expressed genes related the pathway. The red box indicated the up-regulated differentially expressed genes related to flavonoid biosynthesis pathway in resistant cultivars response to *R. solanacearum* at 3 dpi (▲) and 7 dpi (★), respectively.

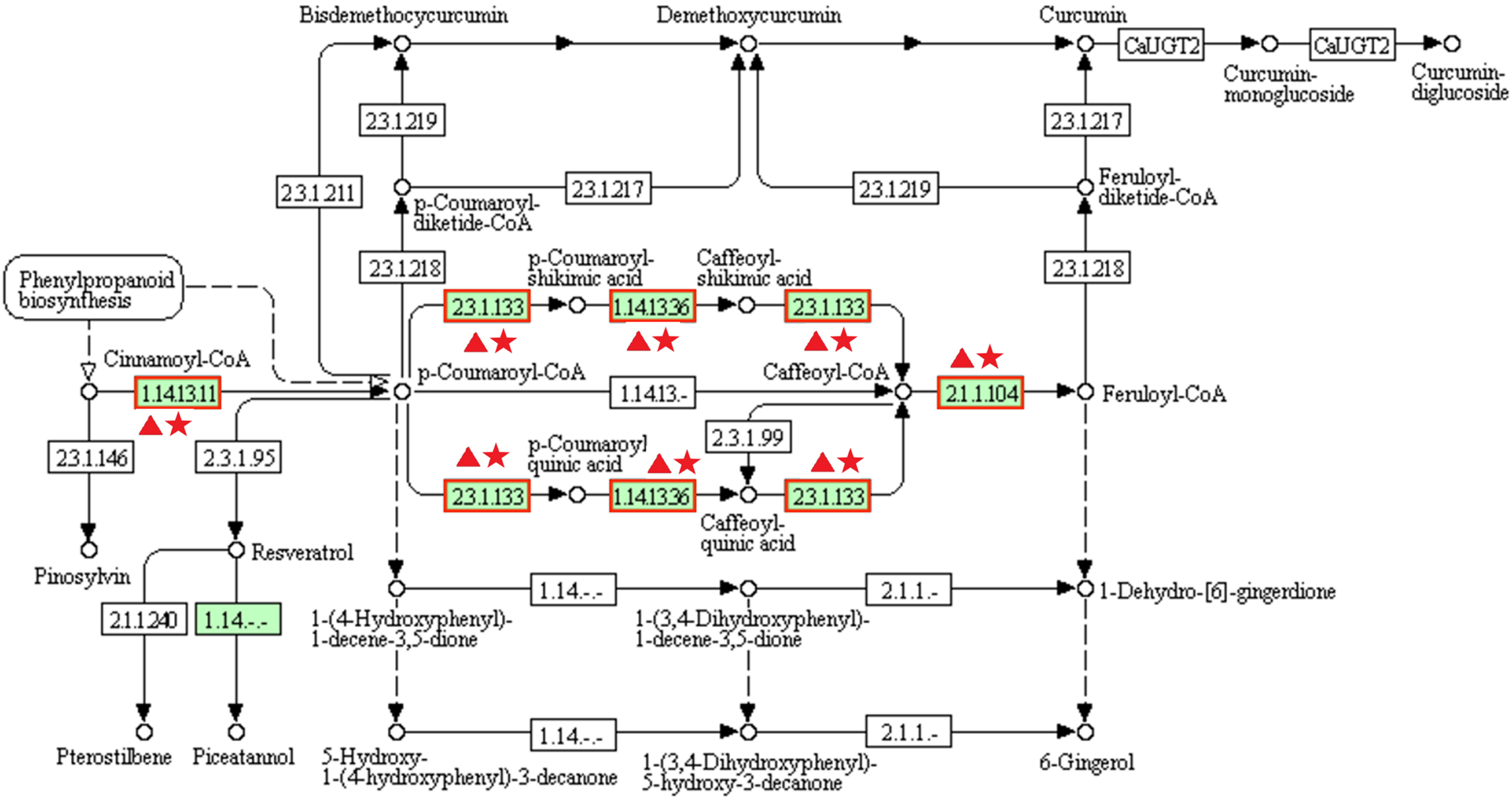

Supplementary Fig. S3. Stilbenoid, diarylheptanoid and gingerol biosynthesis pathway (sly00945) and the differentially expressed genes related the pathway. The red box indicated the up-regulated differentially expressed genes related to Stilbenoid, diarylheptanoid and gingerol biosynthesis pathway in resistant cultivars response to *R. solanacearum* at 3 dpi (▲) and 7 dpi (★), respectively.

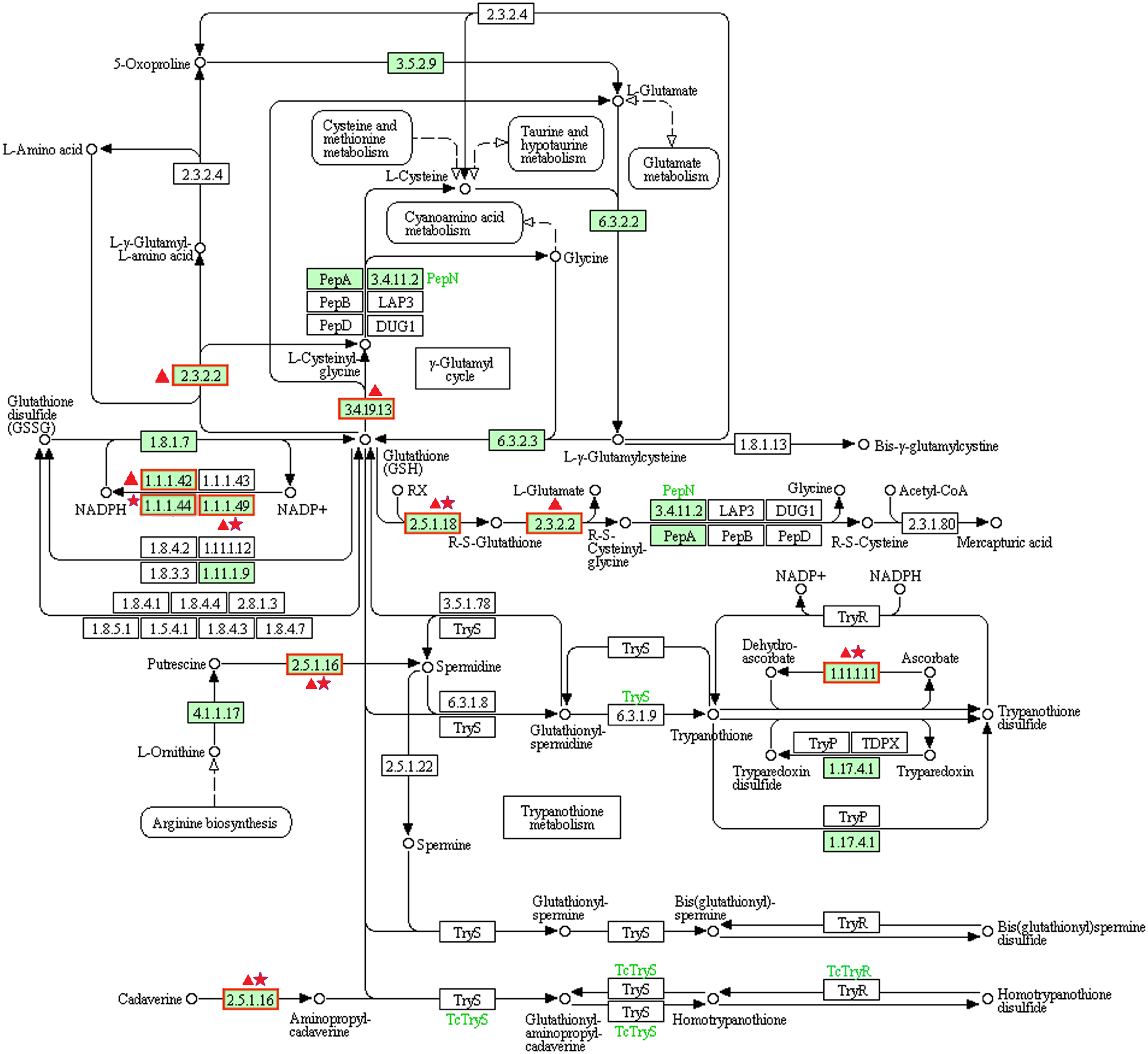

Supplementary Fig.S4. Glutathione metabolism pathway (sly00480) and the differentially expressed genes related the pathway. The red box indicated the up-regulated differentially expressed genes related to Glutathione metabolism pathway in resistant cultivars response to *R. solanacearum* at 3 dpi (▲) and 7 dpi (★), respectively.
