# Supplementary material for: Digital gene expression analysis of the response to Ralstonia solanacearum between resistant and susceptible tobacco varieties
Source: Sci Rep. 2021 Feb 16;11:3887. doi: 10.1038/s41598-021-82576-8 (PMC7886896; doi:10.1038/s41598-021-82576-8)
Supplement: Supplementary file 2 — Supplementary Tables [file 41598_2021_82576_MOESM2_ESM.pdf]

Supplementary Table S1 The information of primer sequences for qRT-PCR

Name	Primer	Sequence	Size
L25	Forward	5’-CCCCTCACCACAGAGTCTGC-3’	51 bp
	Reverse	5’-AAGGGTGTTGTTGTCCTCAATCTT-3’	
gene_23271	Forward	5’-TACCCAAGAGAGCCAGAACC-3’	177 bp
	Reverse	5’-GCTACTCCCTTCTTGCAACT-3’	
gene_34916	Forward	5’-GTGATGATACTGTTATTGGCCT-3’	200 bp
	Reverse	5’-CTCTAAACTTCCTTACCTCCAA-3’	
gene_2681	Forward	5’-AATGTGAACAGCTCAATCTTCC-3’	166 bp
	Reverse	5’-ATCCATTGTACAGGTTTGTGA-3’	
gene_41493	Forward	5’-AGCTCCACAAATGCCTCAGG-3’	154 bp
	Reverse	5’-CTGCCGACCCCAACTTTACT-3’	
gene_11665	Forward	5’-GAACATTTGACACTGCCATA-3’	182 bp
	Reverse	5’-TGAGAGAAACAGTGAATGAG-3’	
gene_64101	Forward	5’-AACGCAAACAGCTCAATCTTCC-3’	220 bp
	Reverse	5’-AAAAAGTCAGGTCACCAGGTG-3’	
gene_31173	Forward	5’-GAAACCACCAGTAGCCGGTAA-3’	203 bp
	Reverse	5’-TCGGCTTCTGCCCGTATTTT-3’	
gene_19445	Forward	5’-GCCGAGGATCTTTCGGGAATA-3’	100 bp
	Reverse	5’-CACCAGTGGGATCTGGGAAG-3’	
gene_56617	Forward	5’-TCAAGGCTGAGCAAGGACAT-3’	229 bp
	Reverse	5’-CCTGATGCTACCAAGGGTTC-3’	

Supplementary Table S2 Statistics of sequencing and reads mapped to the tobacco reference genome in resistant cultivar and susceptible cultivar

Samples	Raw reads	Clean reads	Percent of clean reads to raw reads (%)	Clean bases (G)	Q20 (%)	Q30 (%)	GC content (%)	Total mapped to reference genome	Percent of total mapped (%)
**resistant cultivar**									
In-R1-1	13087224	12965451	99.07	0.65	98.33	95.14	43.47	12342352	95.19
In-R1-2	13593667	13566357	99.80	0.68	98.38	95.28	43.02	13059434	96.26
In-R3-1	12189485	12143471	99.62	0.61	98.34	95.17	43.00	11664800	96.06
In-R3-2	10898324	10870010	99.74	0.54	98.41	95.33	43.12	10424246	95.90
In-R7-1	10254682	10221203	99.67	0.51	98.03	94.29	42.93	9665144	94.56
In-R7-2	12612015	12538376	99.42	0.63	98.31	95.16	42.41	11881326	94.76
M-R1-1	14258457	14233302	99.82	0.71	98.36	95.22	43.09	13726281	96.44
M-R1-2	12535957	12511486	99.80	0.63	98.38	95.28	43.15	12052693	96.33
M-R3-1	14470801	14152957	97.80	0.71	98.50	97.01	43.57	13650985	96.45
M-R3-2	12430186	12160926	97.83	0.61	98.52	97.05	43.42	11774513	96.82
M-R7-1	12531789	12203335	97.38	0.61	98.56	97.13	43.83	11834369	96.98
M-R7-2	11885472	11673175	98.21	0.58	98.49	96.98	43.57	11289127	96.71
**susceptible cultivar**									
In-S1-1	13454878	13136435	97.63	0.66	99.15	97.09	43.15	12705453	96.72
In-S1-2	12782977	12375071	96.81	0.62	99.15	97.08	43.07	11991788	96.90
In-S3-1	11710676	11610179	99.14	0.58	98.89	96.27	43.00	11214833	96.59
In-S3-2	11351851	11292696	99.48	0.56	98.38	95.84	42.54	10744827	95.15
In-S7-1	12121760	12099126	99.81	0.60	98.23	94.82	42.98	11439276	94.55
In-S7-2	12622861	12509911	99.11	0.63	98.23	94.86	42.39	12019459	96.08
M-S1-1	13550512	13522671	99.79	0.68	97.61	93.36	42.97	13019166	96.28
M-S1-2	14393220	14357152	99.75	0.72	97.61	93.39	43.05	13795914	96.09
M-S3-1	13117738	13048488	99.47	0.65	98.49	95.43	42.67	12360458	94.73
M-S3-2	11085033	10903536	98.36	0.55	98.92	96.57	42.81	10510048	96.39
M-S7-1	11339220	11296643	99.62	0.56	98.02	94.26	42.67	10820834	95.79
M-S7-2	11394407	11329489	99.43	0.57	98.38	95.29	42.86	10917629	96.36

The last number at the end of sample name represented duplicate numbers.

Supplementary Table S3 The expression of differential expressed genes related with phenylpropane pathways in resistant cultivar and susceptible cultivar inducing by *R. solanacearum* infection

Gene_id	Resistant cultivar (Fandi 3)		Susceptible cultivar (Yunyan 87)		Function annotation
	log_2 (In/M)	*P*-adj	log_2 (In/M)	*P*-adj	
**3 dpi**					
gene_23271	2.217	0.003	-0.147	1.000	Caffeoyl-CoA O-methyltransferase (CCoAOMT)
gene_31765	0.871	0.022	0.152	1.000	Caffeoyl-CoA O-methyltransferase (CCoAOMT)
gene_61279	1.660	1.103E-06	-0.289	1.000	Caffeoyl-CoA O-methyltransferase (CCoAOMT)
gene_61281	1.495	0.001	0.176	1.000	Caffeoyl-CoA O-methyltransferase (CCoAOMT)
gene_76120	1.388	0.001	-0.158	1.000	Caffeoyl-CoA O-methyltransferase (CCoAOMT)
gene_33653	1.531	8.437E-05	0.553	1.000	Trans-cinnamate 4-monooxygenase (TCM)
gene_34041	1.803	2.381E-09	0.556	1.000	Trans-cinnamate 4-monooxygenase (TCM)
gene_62870	1.704	0.028	1.047	1.000	Trans-cinnamate 4-monooxygenase (TCM)
gene_67175	1.104	0.011	0.669	1.000	Trans-cinnamate 4-monooxygenase (TCM)
gene_13056	1.189	0.008	0.074	1.000	Cytochrome P450 (CYP450)
gene_34916	2.220	1.419E-11	0.141	1.000	Cytochrome P450 (CYP450)
gene_38733	1.343	9.938E-05	0.319	1.000	Cytochrome P450 (CYP450)
gene_42881	1.909	8.347E-09	0.519	1.000	Cytochrome P450 (CYP450)
gene_6890	1.914	2.097E-08	-0.175	1.000	Cytochrome P450 (CYP450)
gene_8682	0.793	0.036	0.162	1.000	Cytochrome P450 (CYP450)
gene_34078	1.171	0.001	-0.088	1.000	Shikimate O-hydroxycinnamoyltransferase (HST)
gene_76735	1.509	0.005	-0.010	1.000	Shikimate O-hydroxycinnamoyltransferase (HST)
gene_1007	4.401	0.030	1.118	1.000	Peroxidase (POD)
gene_21950	4.179	0.019	0.732	1.000	Peroxidase (POD)

gene_38094	4.387	1.953E-06	1.270	1.000	Peroxidase (POD)
gene_48084	1.848	0.036	1.135	1.000	Peroxidase (POD)
gene_58827	3.910	0.022	-0.696	1.000	Peroxidase (POD)
gene_65272	1.182	4.184E-04	0.178	1.000	Peroxidase (POD)
gene_83614	5.383	8.765E-10	1.202	1.000	Peroxidase (POD)
gene_15825	1.581	0.020	0.348	1.000	Phenylalanine ammonia-lyase (PAL)
gene_2681	1.751	8.273E-09	-0.152	1.000	Phenylalanine ammonia-lyase (PAL)
gene_64101	2.015	2.734E-06	0.544	1.000	Phenylalanine ammonia-lyase (PAL)
gene_79790	1.786	0.001	-0.223	1.000	4-coumarate--CoA ligase (4CL)
gene_82065	0.906	0.012	0.402	1.000	4-coumarate--CoA ligase (4CL)
gene_28100	1.083	0.014	-0.365	1.000	Beta-glucosidase (BGL)
gene_79092	1.735	2.440E-05	0.182	1.000	Beta-glucosidase (BGL)
gene_42129	1.017	0.007	-0.120	1.000	Hydroxygeraniol dehydrogenase (HGO)
gene_46557	1.986	1.405E-09	-0.432	1.000	Cinnamoyl-CoA reductase (CCR)
gene_54798	1.875	9.513E-08	-0.357	1.000	Cinnamoyl-CoA reductase (CCR)
gene_50641	1.850	8.554E-07	0.740	1.000	Caffeoylshikimate esterase (CSE)
gene_56734	1.156	0.002	0.258	1.000	Caffeoylshikimate esterase (CSE)
gene_66975	1.842	2.115E-08	0.735	1.000	Cinnamyl alcohol dehydrogenase (CADH)
gene_68771	1.066	0.001	-0.200	1.000	Probable mannitol dehydrogenase (MTDH)
**7 dpi**					
gene_45893	2.590	0.010	0.391	1.000	Chalcone--flavonone isomerase (CFI)
gene_1089	6.165	0.005	2.771	1.000	Caffeoyl-CoA O-methyltransferase (CCoAOMT)
gene_14815	6.223	9.677E-16	1.607	1.000	Caffeoyl-CoA O-methyltransferase (CCoAOMT)
gene_17913	6.413	6.185E-06	2.239	1.000	Caffeoyl-CoA O-methyltransferase (CCoAOMT)

gene_23271	3.696	6.175E-12	1.065	1.000	Caffeoyl-CoA O-methyltransferase (CCoAOMT)
gene_31769	4.255	0.013	0.939	1.000	Caffeoyl-CoA O-methyltransferase (CCoAOMT)
gene_61279	2.986	9.988E-08	0.069	1.000	Caffeoyl-CoA O-methyltransferase (CCoAOMT)
gene_65433	3.165	2.004E-07	1.864	1.000	Caffeoyl-CoA O-methyltransferase (CCoAOMT)
gene_33653	3.689	1.288E-04	-0.101	1.000	Trans-cinnamate 4-monooxygenase (TCM)
gene_34041	4.093	0.003	0.261	1.000	Trans-cinnamate 4-monooxygenase (TCM)
gene_62870	6.810	0.003	1.964	1.000	Trans-cinnamate 4-monooxygenase (TCM)
gene_67175	5.674	0.001	1.191	1.000	Trans-cinnamate 4-monooxygenase (TCM)
gene_29405	2.915	0.022	1.791	1.000	Shikimate O-hydroxycinnamoyltransferase (HST)
gene_13056	1.577	0.033	2.235	1.000	Cytochrome P450 (CYP450)
gene_34916	4.987	0.001	0.440	1.000	Cytochrome P450 (CYP450)
gene_38733	4.780	0.011	0.467	1.000	Cytochrome P450 (CYP450)
gene_42881	5.113	6.357E-20	0.664	1.000	Cytochrome P450 (CYP450)
gene_8682	3.177	3.682E-09	0.823	1.000	Cytochrome P450 (CYP450)
gene_1446	3.304	0.049	0.027	1.000	Peroxidase (POD)
gene_20616	6.887	0.049	3.092	0.250	Peroxidase (POD)
gene_23364	8.690	0.004	8.545	0.315	Peroxidase (POD)
gene_25953	5.158	0.006	0.651	1.000	Peroxidase (POD)
gene_53170	5.992	0.001	5.158	0.280	Peroxidase (POD)
gene_62798	4.844	0.017	3.272	0.481	Peroxidase (POD)
gene_77097	4.931	0.015	3.980	0.429	Peroxidase (POD)
gene_79349	5.645	6.499E-05	1.933	1.000	Peroxidase (POD)
gene_80731	4.155	4.954E-13	1.027	1.000	Peroxidase (POD)
gene_15825	6.560	2.598E-14	0.866	1.000	Phenylalanine ammonia-lyase (PAL)

gene_2681	5.369	3.212E-11	0.440	1.000	Phenylalanine ammonia-lyase (PAL)
gene_57109	2.905	1.896E-07	0.191	1.000	Phenylalanine ammonia-lyase (PAL)
gene_64101	5.679	2.729E-12	0.959	0.976	Phenylalanine ammonia-lyase (PAL)
gene_75046	3.555	9.744E-09	0.116	1.000	Phenylalanine ammonia-lyase (PAL)
gene_75196	8.924	3.498E-08	3.019	1.000	Phenylalanine ammonia-lyase (PAL)
gene_76210	4.872	7.595E-17	-0.213	1.000	Phenylalanine ammonia-lyase (PAL)
gene_64712	4.822	2.197E-13	3.744	1.000	Suberization-associated anionic peroxidase (PERX)
gene_71859	2.873	1.117E-05	1.705	1.000	Suberization-associated anionic peroxidase (PERX)
gene_79404	7.029	0.044	3.891	1.000	Suberization-associated anionic peroxidase (PERX)
gene_79790	3.556	0.011	0.761	1.000	4-coumarate--CoA ligase (4CL)
gene_82065	4.579	0.033	0.737	1.000	4-coumarate--CoA ligase (4CL)
gene_14894	1.886	0.007	0.318	1.000	Beta-glucosidase (BGL)
gene_50641	5.677	0.004	1.322	1.000	Caffeoylshikimate esterase (CSE)
gene_56734	4.112	0.004	0.985	1.000	Caffeoylshikimate esterase (CSE)
gene_54798	1.486	0.050	-0.903	1.000	Cinnamoyl-CoA reductase (CCR)
gene_63068	2.567	0.001	-0.324	1.000	Caffeic acid 3-O-methyltransferase (COMT)
gene_8332	2.700	3.525E-07	0.533	1.000	Caffeic acid 3-O-methyltransferase (COMT)
gene_66975	1.755	0.003	-0.333	1.000	Cinnamyl alcohol dehydrogenase (CADH)

In and M in $\log_2$ (In/M) were read count values of this gene in inoculated samples and mock samples, respectively.

Supplementary Table S4 The expression of differential expressed genes related with glutathione metabolism in resistant cultivar and susceptible cultivar inducing by *R. solanacearum* infection

Gene_id	Resistant cultivar (Fandi 3)		Susceptible cultivar (Yunyan 87)		Function annotation
	log_2 (In/M)	*P*-adj	log_2 (In/M)	*P*-adj	
**3dpi**					
gene_10038	2.074	1.527E-14	0.440	1.000	L-ascorbate peroxidase (APX)
gene_10744	1.022	1.594E-02	-1.094	1.000	L-ascorbate peroxidase (APX)
gene_14850	1.305	4.837E-04	1.637	0.004	L-ascorbate peroxidase (APX)
gene_77930	1.216	1.630E-03	0.741	1.000	L-ascorbate peroxidase (APX)
gene_3531	1.405	3.402E-03	-0.494	1.000	L-ascorbate peroxidase (APX)
gene_2875	2.478	1.081E-19	0.574	1.000	L-ascorbate peroxidase (APX)
gene_56617	2.200	3.497E-03	0.095	1.000	L-ascorbate peroxidase (APX)
gene_21223	1.899	1.713E-03	0.009	1.000	L-ascorbate peroxidase (APX)
gene_37356	2.450	4.236E-03	-0.946	1.000	Glutathione S-transferase (GST)
gene_27796	0.870	1.074E-02	-0.969	1.000	Glutathione S-transferase (GST)
gene_19304	3.652	3.454E-04	1.630	1.000	Glutathione S-transferase (GST)
gene_31648	6.016	2.944E-07	0.165	1.000	Glutathione S-transferase (GST)）
gene_31642	0.767	2.704E-02	0.646	1.000	Glutathione S-transferase (GST)
gene_20284	1.365	1.326E-05	-0.127	1.000	Glutathione S-transferase (GST)
gene_12706	4.153	1.198E-09	3.295	0.005	Glutathione S-transferase (GST)
gene_70102	3.248	7.067E-09	0.835	1.000	Glutathione S-transferase (GST)
gene_24706	0.943	4.720E-02	-0.238	1.000	Glutathione S-transferase (GST)
gene_25955	1.201	3.861E-02	0.270	1.000	Isocitrate dehydrogenase (IDH)
gene_9337	3.984	8.441E-09	-0.298	1.000	Gamma-glutamyltranspeptidase (GGT)
gene_12189	2.118	1.970E-05	0.140	1.000	Gamma-glutamyltranspeptidase (GGT)
gene_56062	2.196	2.078E-03	-0.840	1.000	Glucose-6-phosphate 1-dehydrogenase (G6PD)
gene_39661	1.447	6.067E-07	0.750	1.000	Spermidine synthase (SPDE)
**7 dpi**					
gene_33027	2.807	0.042	0.835	1.000	Spermidine synthase (SPDE)

gene_39661	3.166	0.012	0.822	1.000	Spermidine synthase (SPDE)
gene_58755	6.631	1.274E-16	5.584	1.000	Glutathione S-transferase (GST)
gene_28341	5.823	1.862E-10	5.880	1.000	Glutathione S-transferase (GST)
gene_75845	8.529	2.167E-15	3.325	1.000	Glutathione S-transferase (GST)
gene_64206	2.319	0.007	-0.456	1.000	Glutathione S-transferase (GST)
gene_28342	6.386	0.009	3.227	1.000	Glutathione S-transferase (GST)
gene_67173	7.819	2.619E-08	6.103	1.000	Glutathione S-transferase (GST)
gene_28022	10.499	7.923E-25	7.941	0.897	Glutathione S-transferase (GST)
gene_63998	4.273	0.003	2.770	1.000	Glutathione S-transferase (GST)
gene_37357	5.660	6.585E-19	3.054	1.000	Glutathione S-transferase (GST)
gene_67987	2.477	1.323E-05	1.857	0.866	Glutathione S-transferase (GST)
gene_72914	2.550	8.932E-05	2.783	1.000	Glutathione S-transferase (GST)
gene_28339	2.597	0.037	1.505	1.000	Glutathione S-transferase (GST)
gene_50207	5.870	1.956E-21	5.398	0.976	Glutathione S-transferase (GST)
gene_72905	7.930	0.009	4.311	1.000	Glutathione S-transferase (GST)
gene_72906	9.182	2.120E-04	4.541	1.000	Glutathione S-transferase (GST)
gene_47284	6.445	0.009	2.665	1.000	Glutathione S-transferase (GST)
gene_17386	3.648	0.002	1.010	1.000	Glutathione S-transferase (GST)
gene_30252	8.997	3.054E-33	4.849	1.000	Glutathione S-transferase (GST)
gene_72512	4.616	0.010	1.612	0.948	Glutathione S-transferase (GST)
gene_42369	6.177	8.232E-25	-0.111	1.000	Glutathione S-transferase (GST)
gene_81015	5.538	0.015	3.069	1.000	Glutathione S-transferase (GST)
gene_33938	2.672	0.002	2.622	0.003	Glutathione S-transferase (GST)
gene_12706	4.012	2.613E-08	2.637	0.052	Glutathione S-transferase (GST)
gene_12707	4.376	6.656E-10	1.186	1.000	Glutathione S-transferase (GST)
gene_71544	2.342	0.038	1.637	1.000	Glutathione S-transferase (GST)
gene_71543	5.903	0.002	1.024	1.000	Glutathione S-transferase (GST)
gene_22883	3.159	0.033	2.226	1.000	Glutathione S-transferase (GST)
gene_82831	2.617	3.847E-06	3.046	0.571	Glutathione transferase (GST)
gene_69386	3.737	0.003	3.767	1.000	Glutathione transferase (GST)

gene_10038	1.981	3.073E-04	0.693	1.000	L-ascorbate peroxidase (APX)
gene_2875	2.297	2.011E-05	0.326	1.000	L-ascorbate peroxidase (APX)
gene_56617	2.749	2.453E-07	0.679	1.000	L-ascorbate peroxidase (APX)
gene_21223	7.403	1.916E-34	0.923	1.000	L-ascorbate peroxidase (APX)
gene_16362	1.822	0.013	1.409	1.000	Glucose-6-phosphate 1-dehydrogenase (G6PD)
gene_24989	1.756	0.004	1.281	1.000	6-phosphogluconate dehydrogenase (6PGD)
gene_13084	2.539	6.185E-06	1.460	1.000	6-phosphogluconate dehydrogenase (6PGD)

In and M in $\log_2$ (In/M) was read count values of this gene in inoculated samples and mock samples, respectively.

Supplementary Table S5 The expression of genes related with WRKY transcription factors, ERFs transcription factors and PR in resistant cultivar and susceptible cultivar inducing by *R. solanacearum* infection

Gene_id	3 dpi				7 dpi				Function annotation
	Resistant cultivar (Fandi 3)		Susceptible cultivar (Yunyan 87)		Resistant cultivar (Fandi 3)		Susceptible cultivar (Yunyan 87)		
	$\log_2$(In/M)	*P*-adj	$\log_2$(In/M)	*P*-adj	$\log_2$ (In/M)	*P*-adj	$\log_2$(In/M)	*P*-adj	
**WRKY transcription factors**									
gene_36222	-3.578	0.045	-0.755	1.000	-0.859	0.393	0.237	1.000	WRKY4
gene_11477	3.391	0.014	-0.159	1.000	2.712	0.001	1.608	1.000	WRKY6
gene_2221	4.948	0.005	1.066	1.000	5.807	7.859E-06	0.989	1.000	WRKY6
gene_40867	3.009	0.168	-0.041	1.000	2.916	0.234	5.423	1.000	WRKY6
gene_41493	2.909	0.013	-4.717	0.116	4.232	0.187	0.983	1.000	WRKY6
gene_51838	-0.246	0.689	-0.667	1.000	0.357	0.838	1.209	0.940	WRKY7
gene_78901	0.284	1.000	1.564	1.000	1.357	0.837	3.939	1.000	WRKY9
gene_14657	1.546	0.037	-0.692	1.000	2.412	4.082E-05	-0.165	1.000	WRKY11
gene_31173	2.003	4.301E-06	-0.894	1.000	2.410	1.826E-04	0.178	1.000	WRKY11
gene_33053	1.583	0.193	-0.954	1.000	1.957	0.001	-0.196	1.000	WRKY11
gene_6736	1.658	0.001	-1.192	1.000	2.973	3.193E-05	-0.061	1.000	WRKY11
gene_50556	0.505	0.611	0.409	1.000	1.776	0.694	-3.105	1.000	WRKY13
gene_27371	1.382	0.439	-0.955	1.000	3.304	3.627E-07	2.050	1.000	WRKY15
gene_20456	0.555	0.330	0.550	1.000	1.669	0.009	-0.084	1.000	WRKY19
gene_23939	1.276	1.022E-04	-0.326	1.000	-1.323	0.582	-0.469	1.000	WRKY19
gene_25574	-0.542	0.641	1.166	1.000	0.732	0.686	0.761	1.000	WRKY19

gene_30712	-1.697	4.353E-05	1.593	0.819	-1.121	0.611	0.704	1.000	WRKY19
gene_9313	1.382	7.873E-05	0.154	1.000	-1.387	0.638	-0.305	1.000	WRKY19
gene_25654	1.095	0.070	0.207	1.000	4.569	8.981E-08	-0.424	1.000	WRKY21
gene_7518	1.135	0.015	-0.470	1.000	-0.713	0.466	-0.218	1.000	WRKY23
gene_22514	4.350	0.339	2.126	1.000	2.466	0.301	-1.026	1.000	WRKY28
gene_40423	6.358	1.745E-06	2.103	0.431	1.772	0.322	-0.283	1.000	WRKY28
gene_38545	2.574	0.038	-1.621	0.968	3.319	0.145	3.063	1.000	WRKY33
gene_55876	-0.178	0.809	-1.549	0.004	2.002	0.285	2.596	0.887	WRKY33
gene_83844	1.105	0.344	-2.014	0.655	3.316	0.234	3.326	1.000	WRKY33
gene_32601	-0.840	0.283	-1.513	1.000	2.360	0.088	1.352	1.000	WRKY40
gene_60772	0.682	0.401	-1.496	0.194	2.290	0.001	2.382	1.000	WRKY40
gene_63576	-0.227	0.973	-1.175	1.000	5.180	0.092	1.642	1.000	WRKY40
gene_18725	-0.023	1.000	-1.097	1.000	2.081	0.083	3.122	0.250	WRKY41
gene_20527	3.481	0.254	-1.296	1.000	6.524	9.428E-06	1.584	0.776	WRKY41
gene_60366	2.434	2.249E-06	-1.257	0.534	3.224	0.332	1.510	0.340	WRKY41
gene_63365	0.947	0.068	-0.945	1.000	3.055	0.240	0.648	1.000	WRKY41
gene_12736	0.695	0.980	-1.620	1.000	-2.708	0.623	-0.920	1.000	WRKY42
gene_34812	0.778	0.573	-1.118	1.000	0.098	1.000	1.271	1.000	WRKY42
gene_63855	0.537	0.778	-0.017	1.000	1.780	0.268	2.918	1.000	WRKY42
gene_61257	4.915	3.104E-07	-0.391	1.000	2.788	0.447	-0.906	1.000	WRKY49
gene_25503	2.286	0.467	0.002	1.000	7.498	4.743E-06	3.305	1.120E-04	WRKY50
gene_31899	2.132	0.580	0.296	1.000	7.655	4.472E-04	3.094	0.005	WRKY50
gene_10784	3.043	0.308	0.920	1.000	6.839	0.020	5.784	0.826	WRKY51
gene_17135	1.871	0.672	0.072	1.000	9.393	0.020	4.936	0.555	WRKY51

gene_23574	2.039	0.622	-0.948	1.000	8.519	0.067	5.162	0.377	WRKY51
gene_76938	5.074	0.398	0.192	1.000	9.483	0.057	4.383	0.502	WRKY51
gene_48623	-0.175	1.000	0.771	1.000	3.685	0.081	3.806	0.040	WRKY53
gene_8524	-0.927	0.216	-1.332	1.000	2.575	0.294	3.601	0.011	WRKY53
gene_2694	2.833	0.007	0.538	1.000	2.046	0.516	0.810	1.000	WRKY65
gene_1139	-0.898	0.653	-0.870	1.000	3.921	0.351	5.073	2.012E-11	WRKY70
gene_24248	2.253	0.620	-1.895	1.000	7.548	0.038	4.211	0.363	WRKY70
gene_69209	-0.216	1.000	-1.528	1.000	4.761	0.152	4.474	0.004	WRKY70
gene_71718	1.715	0.622	-0.717	1.000	6.208	0.054	4.214	0.972	WRKY70
gene_77768	0.106	0.994	-0.782	1.000	5.587	0.060	3.270	0.004	WRKY70
gene_79795	-0.024	1.000	-1.069	1.000	4.631	0.223	3.580	0.030	WRKY70
gene_12694	1.846	0.050	0.986	1.000	1.069	0.483	0.552	1.000	WRKY71
gene_9353	1.162	0.851	-1.061	1.000	2.858	0.718	-0.724	1.000	WRKY71
gene_19690	3.903	0.060	2.577	0.334	8.895	0.010	7.959	1.000	WRKY75
gene_41707	5.403	0.169	0.249	1.000	8.154	0.011	4.658	1.000	WRKY75
**ERFs transcription factors**									
gene_79842	-0.761	0.063	-0.786	1.000	-0.093	0.975	-0.100	1.000	ERF1
gene_37385	3.361	0.373	1.865	1.000	3.239	0.218	1.137	1.000	ERF3
gene_43263	1.326	0.017	0.152	1.000	-0.254	1.000	-1.402	0.942	ERF3
gene_11665	3.216	2.078E-06	-2.066	0.089	1.193	0.150	-0.735	1.000	ERF5
gene_65172	-0.026	1.000	-1.737	0.011	0.457	0.619	-0.019	1.000	ERF5
gene_84355	3.112	9.129E-08	-1.025	1.000	3.587	1.004E-05	1.908	1.000	ERF10
gene_1077	-1.181	0.004	-0.603	1.000	-0.721	0.726	-0.153	1.000	ERF11
gene_14860	3.439	3.979E-11	1.378	0.994	2.419	0.041	-0.147	1.000	ERF15

gene_19445	4.042	0.023	1.515	0.667	4.731	0.011	0.021	1.000	ERF15
gene_17518	0.444	0.624	-0.361	1.000	3.778	5.383E-05	1.702	1.000	ERF71
gene_59545	0.419	0.565	-0.206	1.000	2.657	2.093E-06	0.953	1.000	ERF71
gene_69097	-2.323	3.419E-13	0.117	1.000	-1.165	0.314	-0.479	1.000	ERF118
gene_85032	-1.694	5.358E-06	-0.185	1.000	-0.214	0.851	0.136	1.000	ERF118
**PR**									
gene_49643	5.266	0.095	0.500	1.000	5.385	3.419E-04	2.811	1.000	PR STH-2
gene_58059	1.590	0.313	1.042	1.000	5.021	0.390	5.673	1.000	PR STH-2
gene_58070	-1.070	0.475	2.747	0.001	3.549	0.548	0.708	1.000	PR STH-2
gene_71603	2.538	0.490	-2.481	1.000	2.382	0.706	-0.466	1.000	PR STH-2
gene_60017	2.548	0.157	0.433	1.000	3.110	2.866E-08	3.365	1.434E-04	PR-4
gene_61670	5.063	0.445	1.808	0.612	6.026	3.183E-04	7.261	0.479	PR-4
gene_71388	1.295	0.693	-0.629	1.000	2.920	0.048	-3.214	1.000	PR 5
gene_81062	1.325	0.003	-1.254	1.000	1.840	0.624	-3.212	1.000	PR 5

In and M in log_2 (In/M) was read count values of this gene in inoculated samples and mock samples, respectively.
